# The photo-redox of chromium regulated by microplastics (MPs) and MPs-derived dissolved organic matter (MPs-DOM) and the CO_2_ emission of MPs-DOM

**DOI:** 10.1016/j.fmre.2022.08.009

**Published:** 2022-08-24

**Authors:** Enyao Zhang, Yalan Chen, Yang Li, Ke Sun, Yan Yang, Bo Gao, Baoshan Xing

**Affiliations:** a State Key Laboratory of Water Environment Simulation, School of Environment, Beijing Normal University, Beijing 100875, China; b State Key Laboratory of Simulation and Regulation of Water Cycle in River Basin, China Institute of Water Resources and Hydropower Research, Beijing 100038, China; c Stockbridge School of Agriculture, University of Massachusetts, Amherst, MA 01003, USA

**Keywords:** Microplastics, Dissolved organic matter, Chromium, Photo-redox, Mineralization

## Abstract

Microplastics (MPs) and chromium (Cr) are common pollutants in wastewater treatment plants, where ultraviolet disinfection processes may degrade MPs and photooxidize Cr(III) into more hazardous Cr(VI). In this study, the effects of MPs on the phototransformation of coexisting Cr, as well as the role and ecological effects of MPs-derived dissolved organic matter (MPs-DOM), were investigated. The photooxidation of MPs and Cr(III) was radical-driven reaction. The addition of MPs inhibited the photooxidation of Cr(III) and induced the photoreduction of Cr(VI) through surface adsorption. Both MPs and MPs-DOM generated comparable ROS at different irradiation moments, which can affect the photodegradation of MPs and MPs-DOM, as well as the phototransformation of Cr. The 56-day incubation showed a higher mineralization ratio of MPs-DOMs than riverine humic acid, indicating the vital contribution of MPs-DOMs to carbon emissions. In general, MPs-DOM lowered microbial abundance and diversity compared to the original inoculum. Bacterial and fungal succession was affected by both MPs-DOM and the original inoculum, with polyvinyl chloride-derived DOM possessing a stronger filtration impact on microbial communities than the other three MPs-DOMs. In this study, a win–win solution for Cr(VI) reduction and MPs treatment through photoirradiation was proposed, and the potential significant role of MPs-DOM in the transformation of coexisting contaminants, the carbon cycle, and microbial succession was highlighted.

## Highlights

• MPs inhibited photo-oxidation of Cr(III) and induced photo-reduction of Cr(VI).
• MPs and MPs-DOM generated comparable amount of ROS.
• MPs-DOM played an important role in the redox of Cr.
• MPs-DOM is more degradable than riverine humic acid.
• MPs-DOM is a crucial contributor to CO_2_ emission.

## Graphical abstract

**Figure:**
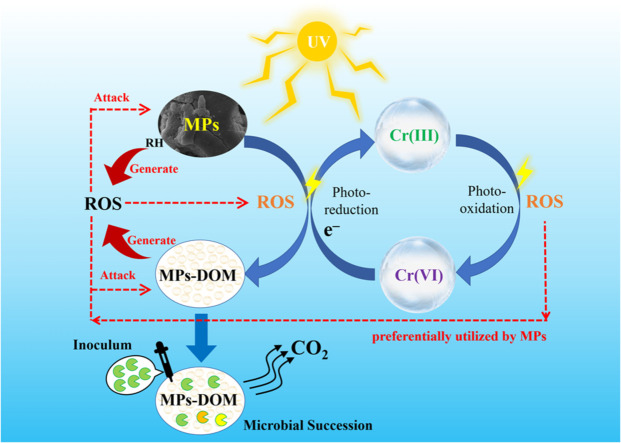

## 1 Introduction

The discharge of industrial effluents from mining operations, metal plating facilities, tanneries, and chromate manufacturing often induces severe chromium (Cr) contamination in aquatic environments [1,2]. Chromium (Cr) pollution often comes out in the form of trivalent chromium (Cr(III)) and hexavalent chromium (Cr(VI)), with the latter exhibiting higher toxicity, and may highly threaten environmental quality and human health [3,4]. In addition, the widely applied ultraviolet (UV) disinfection technology during wastewater treatment may reoxidize residual Cr(III) into more hazardous Cr(VI) [5]. Therefore, finding a way to efficiently alleviate Cr(VI) contamination has become a research priority.

Microplastics (MPs) are another common pollutant in wastewater treatment plants [6]. During the ultraviolet disinfection process, the residual microplastics were plausibly further degraded [7,8]. Such a process may affect the phototransformation of coexisting Cr either as an electron donor or acceptor. Previous scholars have verified the potential of MPs to generate reactive oxygen species (ROS) under photoirradiation [9], which may continuously attack MPs themselves or their coexisting pollutants. Moreover, the photoproducts of MPs dissolved in the aquatic solution, defined here as MPs-derived dissolved organic matter (MPs-DOM) [7,10], may also have the potential to generate ROS as natural DOM and pyrogenic DOM do [11]. To date, while the photodegradation of MPs and generation of MPs-DOMs have received increasing attention, the effects of MPs and MPs-DOMs on the transformation of coexisting pollutants have not been well investigated.

In recent years, an increasing number of studies have focused on the ecological effects of MPs, especially the carbon and nitrogen cycles [12,13]. However, little is known about such ecological effects of MPs-DOM. MPs-DOMs usually comprise small molecules [7,10,14], such as alcohols, acids, aldehydes, ketones and unsaturated groups [15], and may be more easily mineralized than their parent solids. Some studies have also stressed the potential role of natural DOM in the succession of microbial communities [16]. It is hypothesized that MPs-DOMs also possess certain filtration effects on aquatic microbes and continuously affect the aquatic environment during their degradation.

In this study, the Cr(III) solution with or without MPs addition was subjected to photoirradiation, and a short-term incubation experiment of MPs-DOMs was also conducted. The major objectives of this study were to (1) track the photooxidation and reduction dynamics of Cr with the assistance of MPs, (2) verify the role of ROS in the phototransformation of Cr and photodegradation of MPs, (3) reveal the ability of MPs, as well as MPs-DOM, to generate ROS, and (4) examine the short-term mineralization ratio of different MPs-DOMs and the potential impact of MPs-DOMs on microbial succession.

## 2 Materials and methods

### 2.1 Materials

Four analytically pure MPs, including polystyrene (PS), polyamide (PA), polyvinyl chloride (PVC), and polypropylene (PP), were purchased from Youngling-TECH Company (Shanghai, China). These experimental MPs were fragment-shaped and free of additives (e.g., stabilizers, antioxidants or brominated flame retardants). All MPs samples were passed through a 100-mesh sieve and washed alternately with Milli-Q water and absolute ethanol to remove impurities prior to use.

The Cr(III) nitrate nonahydrate (AR, 99.95%) and diphenylcarbazide (AR, ≥ 99.5%) used in this study were purchased from RHAWN reagent. The Cr(VI) standard solution (1000 ug/mL) was purchased from Guobiao (Beijing) Testing & Certification Co., Ltd. The SRHA (Suwannee River Humic Acid Standard III, 3S101H) was purchased from the International Humic Substances Society (IHSS, www.humicsubstances.org). Tert-butyl alcohol (TBA, ≥ 99.5%) and 2,2,6,6-tetramethyl-4-piperidinol (TEMP, ≥ 99%) were purchased from Aladdin (Shanghai, China). *p*-Benzoquinone (PBQ, 99%) was purchased from Shanghai Macklin Biochemical Co., Ltd. Absolute ethanol, acetone (AR, ≥ 99.5%), hydrochloric acid (AR, 36.0-38.0%), and sodium hydroxide (AR, ≥ 96.0%) were purchased from Beijing Chemical Works (China). Other reagents, including 5,5-dimethyl-1-pyrroline N-oxide (DMPO, ≥ 99%) and 2,2,6,6-tetramethyl-1-piperidinyloxy (TEMPO, ≥ 99%), were purchased from Dojindo Laboratories. Milli-Q water was used throughout the experiments.

### 2.2 Phototransformation experiment of Cr and MPs

The photochemical experiments were performed in a PL-DY1600 photochemical reactor (Beijing Precise Technology Co., Ltd., China). For the phototransformation experiment of MPs alone, 0.1 g MPs were mixed with 50 mL Milli-Q water in a 100 mL silica tube and magnetically stirred at 25 rpm using a Teflon magnet. The tube mouth was covered with aluminum foil to avoid extra water evaporation. In a typical run, a maximum of eight silica glass tubes were subjected to photoirradiation with 18 low-pressure mercury ultraviolet lamps (254 nm, 10 W/m^2^) encompassed at a distance of 0.1 m. The eight silica tubes were horizontally rotated about the center at 50 rpm to receive uniform irradiation from surrounding UV lamps. The pH was controlled at pH = 5.5 to avoid Cr(VI) precipitation at higher pH as per a previous study [17]. The reaction temperature was maintained at 25 ± 5°C by a built-in air-cooling system of the instrument. Samples were withdrawn from the photochemical reactor at 0, 1, 2, 4, 8, 16, 24, 40, 72, and 120 h based on the real UV disinfection time in sewage treatment [18], and passed through a 0.45 μm Teflon membrane to separate aged MPs and corresponding MPs-DOMs. The filtrates (MPs-DOMs) were collected and stored at 4 °C for further analysis. The solids (aged MPs) were dried at 25 °C, kept in sealed containers, and stored in a cool and dry place.

The phototransformation experiments of Cr with MPs addition were carried out under similar experimental conditions. In brief, 0.1 g MPs were mixed with 50 mL 2 mg L^‒1^ Cr(III) solutions in a 100 mL silica tube as the experimental group. The rational for choosing this MPs concentration (2 g/L) was based on the reported MPs concentration ranging from 0.00297 to 2.5803 g/L in freshwater bodies [19]. The Cr concentration used was also environmentally related, as the treated tannery wastewater still contains 2–10 mg/L Cr(III) [4]. The control group was treated by subjecting the Cr(III) solutions without MPs addition to photoirradiation or dark treatment. Similar to the aforementioned procedure, samples were withdrawn periodically, and corresponding solids and liquids were collected separately for further analysis. All treatments were performed in triplicate.

### 2.3 Incubation experiments of MPs-DOM

The incubation experiment was designed as a two-factorial of MPs-DOM and inoculum type. MPs-DOMs generated after 40 h irradiation were collected and diluted to ∼20 mg C/L for laboratory incubation. Additionally, SRHA solution with a concentration of ∼20 mg carbon per liter solution (mg C/L) was prepared as a representative of natural DOM. Two inocula were prepared by concentrating the microorganism suspensions from the Yongding River diversion canal and aeration tank sludge in Beijing Longqing Capital Water Co. LTD. The inoculum was concentrated by passing 1 L of microorganism suspension through a 0.22 μm Millipore membrane and eluting the microorganism on the membrane using Milli-Q water.

The experiment was initialized in August 2021 and lasted for 56 days. A total of ten treatments with three replications were conducted. Each 100 mL bottle received 50 mL 20 mg C/L MPs-DOMs and 0.4 mL inoculum, as well as a nutrient supply of ∼20 μM NH_4_^+^ and ∼2 μM PO_4_^3−^ as per a previous study [20]. These bottles were sealed using disposable rubber aluminum caps, and the headspace gas samples were withdrawn periodically after 0, 1, 3, 7, 14, 21, 28, 42, and 56 days of incubation using a 10 mL gas‐tight locking syringe. After the collection of gas samples at different incubation times, the bottle caps were removed for fresh air replacement. Then, the bottles were resealed, and the initial gas samples of the next incubation stage were collected.

### 2.4 Analytical methods

#### 2.4.1 Characterization of MPs and MPs-DOMs

The functional groups of pristine and aged MPs were determined utilizing a Nexus 670 Fourier transform infrared (FTIR) spectrometer (Nicolet Instrument Corporation, Madison, USA) with a wavelength range of 400-4000 cm^‒1^ and a resolution of 4 cm^‒1^. The morphological changes of MPs after 8 h of UV irradiation were characterized using a scanning electron microscope (SEM, Zeiss Gemini300, Germany) equipped with an energy dispersive X-ray analyzer (EDX, EX-350, Hitachi, Japan). The surface elemental compositions of pristine and aged MPs were measured by X-ray photoelectron spectroscopy (XPS, Thermo Scientific K-Alpha+, Thermo Fisher, USA). The surface zeta potential of MPs, an indicator of their stability and dispersibility in water [21], was monitored using a NanoBrook Omin instrument (Brookhaven Instruments Corporation, New York, USA).

The dissolved organic carbon concentrations (DOC) of MPs-DOMs were determined using a total organic carbon analyzer (TOC-L CPN, Shimadzu, Japan) equipped with an ASI-L autosampler. The ultraviolet absorbance spectra of MPs-DOM were recorded using UV–vis spectroscopy (DR6000) with a scanning range of 200-800 nm and a step size of 1 nm. The SUVA_254_ value (ratio of absorbance at 254 nm to DOC concentration) was utilized as the indicator of MPs-DOM aromaticity [22]. The fluorescence spectroscopy (F-7000, Hitachi, Japan) was applied to obtain the three-dimensional excitation-emission matrices (EEM) of MPs-DOM. The excitation (Ex) and emission (E_m_) wavelengths were set as 200–500 and 250–550 nm with 5 nm steps, respectively. The molecular composition of MPs-DOM pre- and post-microbial incubation was determined using the EI scan mode of SCIONSQ-456GC–MS.

#### 2.4.2 Detection of Cr(III) and Cr(VI) concentrations

The concentrations of Cr(III) and Cr(VI) in aqueous solution were measured as follows. First, UV–vis spectroscopy was used to quantify Cr(VI) concentrations in solution by the diphenylcarbazide colorimetric method at 540 nm with a detection limit of 0.004 mg/L. Then, the total Cr concentrations in the solutions were determined using an inductively coupled plasma optical emission spectrometer with a detection limit of 0.03 mg/L (ICP–OES, iCAP 7000, Thermo Fisher Scientific, USA). Finally, the Cr(III) concentration was obtained by subtracting the Cr(VI) concentration from the total Cr concentration [23].

To determine the Cr(III) and Cr(VI) concentrations adsorbed on MPs surface, 0.1 M HCl and KH_2_PO_4_ were used to desorb the Cr ions from the separated MPs particles [23,24]. The desorption liquid was collected to determine the concentration of absorbed Cr(III) and Cr(VI) ions.

#### 2.4.3 ROS identification and quenching experiment

MPs particles and MPs-DOM collected at different irradiation moments were coupled with DMPO (50 mM), BMPO (30 mM), and TEMP (10 mM) to capture specific ROS and measured by electron spin resonance spectroscopy (ESR, JES-FA100, Japan) [25]. In this study, DMPO was used as a capture agent for •OH, and the spin adduct (DMPO-•OH) featured the quadruple characteristic signal peaks 1:2:2:1; BMPO was used to capture •OH and O_2_^•−^; TEMP was the capture agent for ^1^O_2_, and the spin adduct (TEMP-^1^O_2_) showed a triple characteristic peak of 1:1:1. The EPR instrument parameters were set as follows: center field, 336.0 mT; microwave power, 4.0 mW; sweep width, 5 G; time constant, 0.1 s; sweep time, 1 min; and frequency, 9441 MHz.

For the detection of ROS generated from MPs, 1 mg of MPs was mixed with 50 μL of capture agent in a 1.5 mL centrifuge tube. The MPs particles were dispersed evenly after 20 minutes of sonication and siphoned into the capillary spotting tube. Then, the capillary spotting tube was subjected to UV irradiation for a specific time (60 s for DMPO and TEMP adducts and 45 s for BMPO adducts), and these treated sample tubes were immediately inserted into the ESR cavity for testing. For the detection of ROS generated from MPs-DOM, 25 μL of MPs-DOM solution and 25 μL of capture agent were mixed in a 1.5 mL centrifuge tube and shaken for 2 minutes to make the mixture uniform. The following steps were the same as the ROS measurement procedure for MPs. The controls were performed by subjecting the UV-treated aqueous solution containing solely capture agents to ESR measurements. All the measurements were conducted in the same batch to provide semiquantitative comparisons.

Quenching experiments were also performed to explore the role of ROS in the phototransformation of MPs and Cr. TBA was used to quench •OH, and PBQ was used as a scavenger for O_2_^•−^ and ^1^O_2_. The mass recovery of MPs was measured to probe the role of specific ROS in the degradation of MPs. The Cr(III) and Cr(VI) concentrations at different irradiation moments were measured to identify the effects of ROS on the oxidation and reduction of Cr.

#### 2.4.4 Measurements of cumulative CO_2_ emissions

The CO_2_ concentrations of all gas samples were measured immediately after gas collection using a gas chromatograph (Agilent 7890B) equipped with a flame ionization detector (FID, 250 °C). The cumulative CO_2_ emissions were calculated by subtracting the initial CO_2_ concentration from the final CO_2_ concentration after a period of incubation.

The cumulative CO_2_ emissions of MPs-DOM were fitted to a two-pool exponential model:

(1) $$M_{DOM}\;=\;M_{L}\;\left(1\;--\;e^{-k_{1}t}\right)\;+\;\left(100\;-\;M_{L}\right)\;\left(1\;-\;e^{-k_{2}t}\right)$$

where *M_DOM_* is the cumulative mineralization percentage of MPs-DOMs (%), *M_L_* and (100−*M_L_*) are the proportion of labile and recalcitrant DOM pool in total MPs-DOMs (%), *t* is the incubation time (d), and *k*_1_ and *k*_2_ are the biodegradation rate constants for the labile and recalcitrant carbon (d^−1^).

The half-lives of the labile and recalcitrant DOM pools were calculated as follows:

(2) $$\text{Half}-\text{life}=\ln\left(2\right)\times k^{-1}$$

where *k* is the biodegradation rate constant for labile or recalcitrant carbon (d^−1^).

#### 2.4.5 DNA extraction and sequencing

Both the initial inocula and the incubated MPs-DOM solutions were subjected to DNA extraction using M9. Equalbit 1x dsDNA HS Assay Kit (Lot: 7E551E1) and M1. Magen Hipure Soil DNA Kit (Lot: HD150300). The concentration of DNA was monitored using a Qubit® dsDNA HS Assay Kit. The next-generation sequencing library was constructed using a MetaVX Library Preparation Kit (Azenta, Inc., South Plainfield, NJ), and sequencing was performed on an Illumina MiSeq/Novaseq Platform (Illumina, San Diego, USA). For bacterial 16S rDNA sequencing, a series of proprietary primers was designed to amplify the V3 and V4 hypervariable regions. The forward primers contained the sequence CCTACGGRRBGCASCAGKVRVGAAT, while the reverse primers contained the sequence GGACTACNVGGGTWTCTAATCC. For fungal ITS rDNA sequencing, amplicons of the hypervariable ITS2 region were obtained using forward primers containing the sequence GTGAATCATCGARTC and reverse primers containing the sequence GTGAATCATCGARTC. The follow-up data analyses were performed as per a previous study [26]. All sequence data were deposited in the National Center for Biotechnology Information (NCBI) under accession number PRJNA800179.

### 2.5 Statistical analyses

Statistical analyses, including Pearson correlation analysis, one-way analysis of variance (ANOVA), and three-dimensional principal coordinate analysis (3D-PCoA), were processed and visualized using IBM SPSS 22.0 (SPSS Inc. Chicago, IL, USA) and OriginPro 2019b (OriginLab).

## 3 Results and discussion

### 3.1 Photodegradation of MPs and production of MPs-DOM: Role of ROS

**Fig. 1:**
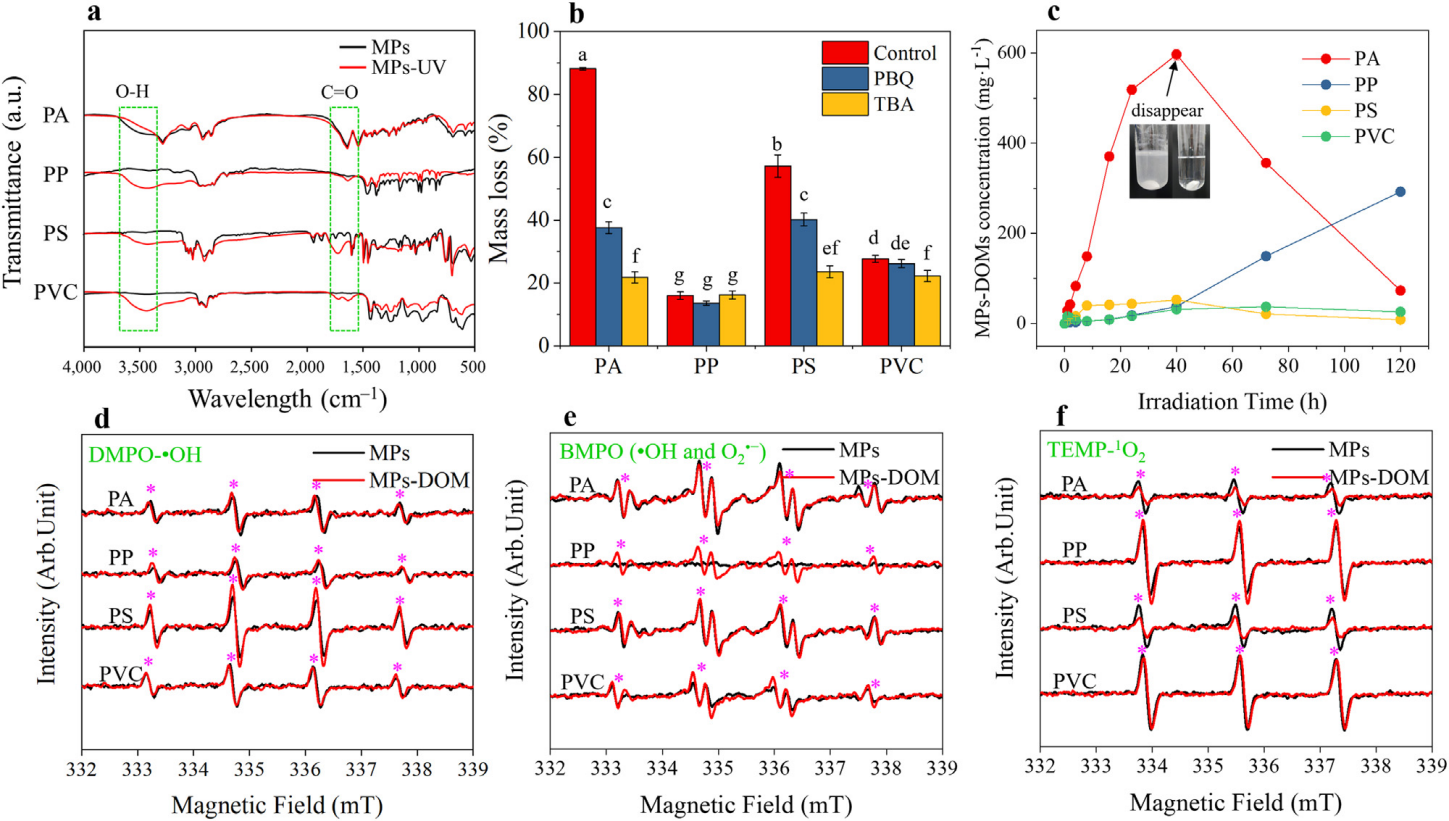
**Characteristics of MPs and MPs-DOM under UV irradiation**. (a) Changes in the functional groups of MPs before and after 8 h of UV irradiation. (b) Mass loss of four experimental MPs after 40 h UV irradiation using different quenching agents. (c) Changes in MPs-DOM concentrations released from the four experimental MPs as irradiation time increased. (d-f) Intensity of DMPO spin adduct (DMPO-•OH) (d), BMPO spin adduct (e), and TEMP spin adduct (TEMP-^1^O_2_) (f) generated by MPs and corresponding MPs-DOMs after 8 h UV irradiation according to semiquantitative ESR spectra. The ESR spectra of DMPO, BMPO and TEMP spin adducts at different moments during the whole irradiation process are shown in Fig. S4. Different lowercase letters represent significant differences between different treatments (*p* < 0.05).

Photoirradiation greatly modified the physicochemical properties of MPs in aquatic environments [14,[27], [28], [29]], including the color, surface morphology, zeta potential, and functional groups (Figs. 1a and S1). As photooxidation proceeded, the colors of PS and PVC progressively turned yellow and brown, respectively, while those of PP and PA were minimally altered (Fig. S1a). Varying degrees of cracks and pits occurred on the surface of different MPs (Fig. S1b), which has also been reported in previous scholars [30]. Additionally, photoirradiation slightly decreased the negative surface potential of PA, PVC, and PP but greatly increased that of PS, suggesting a decreasing stability and dispersibility of MPs in water, except for PS (Fig. S1c). Similar results can also be observed in previous studies [9,21]. Moreover, the characteristic peaks of O-H and C=O stretching were newly formed in aged PS (3434, 1721 cm^−1^), PVC (3445, 1717 cm^−1^) and PP (3442, 1636 cm^−1^) after irradiation (Fig. 1a) [31], [32], [33], [34], suggesting the oxidation of these three MPs under photoirradiation. Similarly, more O-containing functional groups were observed on the surface of aged PS according to XPS spectra (Fig. S1d).

Photoirradiation also resulted in sharp decreases in the mass of all MPs (Fig. 1b). Although the functional groups of PA were minimally altered under photoirradiation, the mass of PA was most profoundly reduced. After 40 h of photoirradiation, PA almost completely disappeared (Fig. 1b, c). The mass loss of MPs followed a descending order of PA (88.2%) > PS (57.2%) > PVC (27.7%) > PP (16%) at 40 h (Fig. 1b), suggesting that PA and PS was more photolabile. The difference in degradability might be related to polymer aromaticity, as the absorption of UV radiation by the aromatic ring in PA and PS may initialize the photodegradation reaction [35,36]. In comparison, PP doesn't contain chromophores in its regular structure and is semi-crystalline. The crystallinity of PP regulates the diffusion of oxygen, which occurs only in the amorphous phase [37]. Thus, the photoreactivity of PP was far weaker than those of PA and PS. With respect to PVC, photoirradiation results in the generation of HCl through the dehydrochlorination process, which has harmful effects on the polymeric chains [38]. This can probably explain the higher degradation ratio of PVC than PP. The quenching experiment showed that the mass loss of MPs was reduced by using TBA or PBQ as a quenching agent for •OH or O_2_^•−^ and ^1^O_2_ [9], except for PP quenched by TBA. This was also supported by a previous study showing that O_2_^•−^ rather than •OH dominated the photoaging of PP in seawater [39]. Overall, the results illustrated that ROS played a significant role in MPs degradation under photoirradiation.

As photodegradation proceeded, varying concentrations of DOM were released from different MPs (Fig. 1c) [7]. In the initial 40 h, the DOM concentrations of the four MPs all increased as the irradiation time increased. The DOM release rate followed a descending order of PA >> PS > PP > PVC. It is reasonable to speculate that the DOM release rate of MPs is related to their aromatic structure, which may adsorb the UV radiation and initialize the photodegradation reaction [35,36]. The low DOM release of PVC was potentially attributed to the low carbon proportion in PVC solid (38.7% ± 0.7%) than other polymers (63.5% ± 0.4%‒91.8% ± 0.5%) (Table S1). When the irradiation time exceeded 40 h, the DOM concentration of PP further increased, while that of PA, PS, and PVC exhibited a decreasing trend. PA-DOM showed the sharpest decrease, indicating the quick decomposition of PA-DOM as the PA solid disappeared. The SUVA_254_ values of PS and PA first increased and then decreased with continuous irradiation, suggesting the initial generation of aromatic photoproducts in DOM solutions but subsequent decomposition of the aromatic fractions as the irradiation time increased (Fig. S2). Only small changes were observed in the SUVA_254_ values of PVC-DOM and PP-DOM, which was attributed to the generation of few aromatics during the whole process. During the photooxidation process, PS and PA also generated some fluorescent photoproducts in the DOM solutions, while PVC and PP showed almost no characteristic fluorescence peaks throughout the whole process (Fig. S3). The fluorescent intensity and area of PS-DOM and PA-DOM first increased and then decreased, the overall trend of which was consistent with those of SUVA_254_ values. The fluorescent fractions of PS-DOM were associated with protein/phenol-like substances [10,40], while those of PA-DOM corresponded to marine humic-like compounds and large molecular-sized and hydrophobic compounds [41,42]. After 120 h of irradiation, no fluorescent components were observed in any MPs-DOM.

As mentioned above, the photodegradation of MPs is driven by ROS, and MPs can efficiently generate MPs-DOM. Thus, the ability of MPs-DOM to generate ROS was further investigated. Interestingly, MPs and MPs-DOM produced comparable amounts of •OH, O_2_^•−^ and ^1^O_2_ at different irradiation moments (Figs. 1d‒f and S4). After 8 h of photoirradiation, the ability of MPs to generate •OH, O_2_^•−^ and ^1^O_2_ followed a descending order of PS > PA > PVC > PP, PA > PS > PVC > PP, and PP > PVC > PS > PA, respectively (Fig. 1d‒f). A consistent order of ROS generation ability can be observed in the corresponding MPs-DOM. The intensity sequence of the BMPO spin adduct generated by MPs was in compliance with the mass loss of the corresponding MPs (Fig. 1b, e), partially indicating the vital role of •OH and O_2_^•−^ in the degradation of MPs. Moreover, PP generated the most ^1^O_2_ but degraded the least, suggesting that •OH and O_2_^•−^ were probably the main oxidants for the degradation of MPs compared to ^1^O_2_ (Fig. 1d‒f). In addition, the intensity sequence of the BMPO spin adduct generated by MPs-DOMs was consistent with the concentration of the corresponding MPs-DOMs (Fig. 1c). Additionally, the intensity sequence of DMPO and BMPO spin adducts generated by MPs-DOMs varied in accordance with the concentration shifts of MPs-DOMs at different irradiation moments (Fig. S4a, b). This is understandable, as DOM at higher concentrations had a stronger ability to generate ROS [43]. A similar sequence can be observed in the intensity of the DMPO spin adduct except that PS and PS-DOM generated higher concentrations of •OH than PA and PA-DOM (Fig. 1d). One possible explanation is that the persistent free radicals generated by PS rather than PE or PVC fascinated the production of more •OH [44]. Overall, the results suggested that MPs-DOM contributed to the generation of ROS, which may facilitate the degradation of MPs particles or the further decomposition of their photoproducts.

### 3.2 Radical-driven photooxidation of Cr(III) and MPs-induced photoreduction of Cr(VI)

**Fig. 2:**
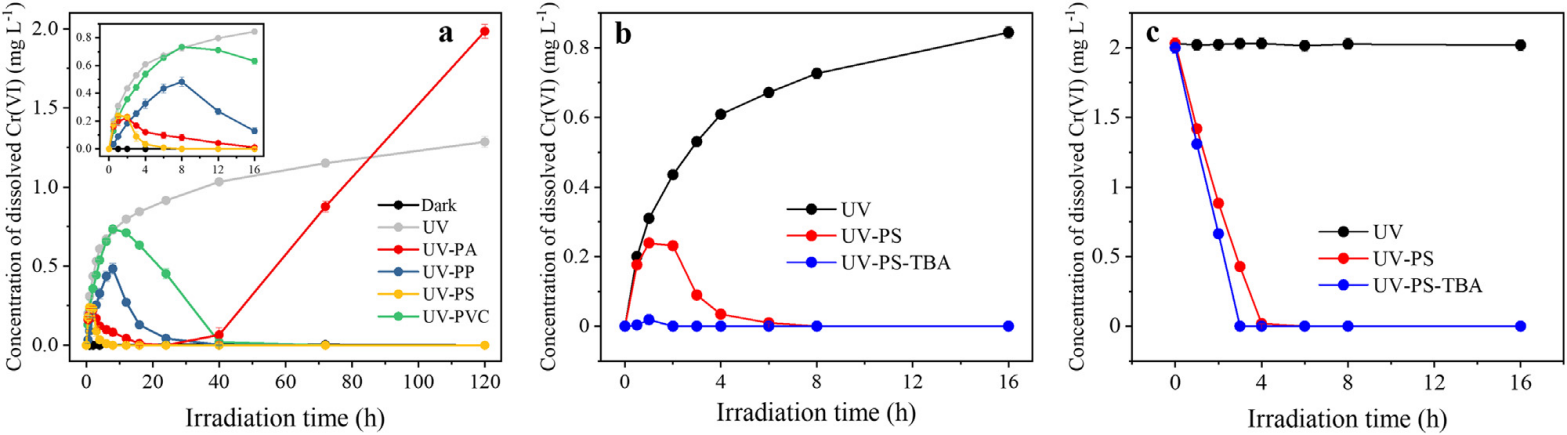
**Shifts in the concentration of dissolved Cr(VI) by MPs addition (a) and TBA quenching agent application (b-c) at different irradiation moments**. The initial Cr(III) (a-b) or Cr(VI) (c) concentration was 2 mg L^–1^. The UV and dark controls were prepared by subjecting Cr(III) solutions without MPs addition to photoirradiation or dark treatment. The inset is the amplification of the dynamics of Cr(VI) generation during 0-16 h.

The presence of MPs significantly modified the transformation between Cr(III) and Cr(VI) under UV irradiation (Fig. 2a). For the dark control, Cr(III) was not oxidized to Cr(VI), and Cr(VI) was not reduced to Cr(III) with MPs addition. In contrast, the concentration of photogenerated Cr(VI) gradually increased with the extension of irradiation time. The photooxidation of Cr(III) conformed to the pseudosecond-order kinetic model. Interestingly, the addition of MPs inhibited the photooxidation process of Cr(III) but drove the photoreduction of Cr(VI) under UV irradiation. The whole phototransformation process can be divided into three stages: 1) the initial photooxidation process; 2) the MPs-induced photoreduction process; and 3) the reoxidation of Cr(III) after MPs disappeared.

In the initial photooxidation stage, the oxidation from Cr(III) to Cr(VI) experienced varying degrees of inhibition by the addition of different MPs (Fig. 2a). This is understandable, as the addition of MPs competed for the utilization of the ROS and thus inhibited the photooxidation of Cr(III), while the photooxidation of Cr(III) was driven by ROS as indicated by the quenching experiment (Fig. 2b). The photooxidation rate of Cr(III) followed a descending order of PS > PA > PVC > PP, which was consistent with the intensity sequence of •OH and O_2_^•−^ generated from different MPs (Figs. 1d, e, and 2a), suggesting that the photooxidation of Cr(III) may be associated with ROS-driven reactions. The maximum concentrations of photoproduced Cr(VI) had the order of PVC > PP > PS > PA.

In the second stage, the subsequent photoreduction of Cr(VI) occurred after irradiation times of 1, 2, 8 and 8 h for PS, PA, PP and PVC, respectively, suggesting faster startup of the photoreduction of Cr(VI) by PS and PA than PP and PVC, although the former generated more ROS (Fig. 2a). This indicated that the self-generated ROS may first initialize the photooxidation of Cr(III) and then be preferentially utilized by different MPs.

In the third stage, the PA solid completely disappeared, and Cr(III) was reoxidized into Cr(VI) (Figs. 1c and 2a). The photooxidation rate of Cr(III) in PA solution during the third stage was smaller than that during the first stage, which was potentially attributed to the generation of less •OH and O_2_^•−^ by solely PA-DOM during the third stage (Fig. S4). Surprisingly, the final Cr(VI) concentration in PA solution even exceeded that in solely Cr(III) solution without MPs addition after 120 h irradiation (Fig. 2a). One potential explanation is that the photoproducts of PA may continuously serve either as an electron donor or acceptor and raise the oxidation limits of Cr(III) to Cr(VI). Also, the highly reactive radical •OH radicals generated by NO_3_^−^ photolysis may contribute to the photooxidation of Cr(III) [45]. It is plausible to speculate that the rest three polymers may also turn to promote the photooxidation of Cr(III) after being extensively photodegraded, as the phototransformation of their photoproducts may facilitate the photooxidation of Cr(III) to Cr(VI).

The quenching experiment further underlined the potential mechanisms for the photoreduction of Cr(VI) by MPs (Fig. 2b, c). As the quenching agent PBQ interfered with the detection of Cr concentration, we only investigated the effects of •OH on the phototransformation of Cr using TBA as a quenching agent. The quenching of •OH more profoundly inhibited the photooxidation of Cr(III), indicating that •OH was one of the major driving factors of Cr(III) oxidation (Fig. 2b). In addition, the photoreduction of Cr(VI) was accelerated by quenching •OH (Fig. 2b, c). The results suggested that •OH contributed to the photooxidation of Cr(III) and exhibited weak inhibiting effects on the photoreduction of Cr(VI) (Fig. 2c). It is worth noting that both MPs and MPs-DOM generated comparable amounts of ROS, indicating that MPs-DOM may also play a role in the phototransformation of Cr(III) and Cr(VI).

**Fig. 3:**
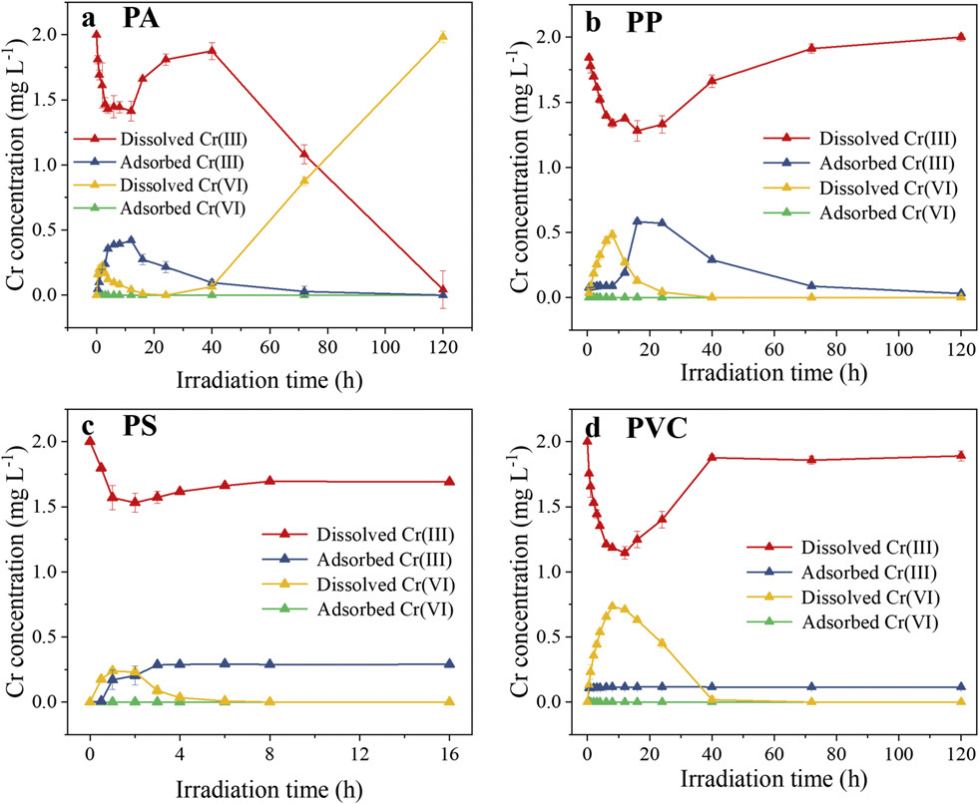
**Shifts in the concentrations of dissolved and adsorbed Cr(III) and Cr(VI) by PA (a), PP (b), PS (c), and PVC (d) with an initial Cr(III) concentration of 2 mg L^–1^**.

The photoirradiation also affected the sorption behavior of Cr on MPs (Fig. 3). Throughout the whole photoirradiation process, hardly any Cr(VI) was adsorbed on MPs, which can be supported by the weak adsorption of Cr(VI) on PS and polyethylene terephthalate [46]. However, the adsorption of Cr(III) on MPs was significantly altered. The absorbed concentration of Cr(III) on PA and PP first increased but then decreased as the photoirradiation time was prolonged (Fig. 3a, b). The increased Cr(III) adsorption was potentially attributed to the photoreduction reaction of Cr(VI) on the surface of MPs. First, Cr(III) competed for the ROS generated on the MPs surface, but most ROS was preferentially utilized for the degradation of MPs and MPs-DOM. Then, the photoproduced Cr(VI) was photoreduced, whereas MPs and MPs-DOM were protoxidized. At irradiation times of 0 and 120 h, pristine and UV-aged PP can adsorb Cr(III), while Cr(III) was not adsorbed on PA. For PS, the adsorbed Cr(III) concentration first increased and then remained at a certain level, which was attributed to the enhanced adsorption ability of UV-aged PS (Fig. 3c). With respect to PVC, the concentration of adsorbed Cr(III) remained at 0.11 mg L^–1^ during the whole process (Fig. 3d). Previous records have shown a higher adsorption capacity of Cr(III) by PVC and PP than PS [47]. Our results further confirmed the order of adsorption capacity as aged PS > PVC = aged PVC > PP > aged PP > PS = PA = aged PA.

The transformation of Cr(III) to Cr(VI) and the degradation of MPs were both photooxidation reactions. Thus, MPs incorporation may be involved in the phototransformation of Cr. Moreover, as the ROS generation potential of natural and pyrogenic DOM has been widely reported [11], it was plausible to speculate that the photoproducts of MPs (i.e., MPs-DOM) could generate ROS. These ROS may attack the MPs and MPs-DOM and affect the phototransformation of Cr. To verify this speculation, the photooxidation and reduction rates of Cr with or without MPs incorporation were compared. The results showed that the photooxidation of Cr(III) experienced varying degrees of inhibition by different MPs. Then, the photoreduction of photoproduced Cr(VI) occurred. The quenching experiment verified that ROS played a leading role in the oxidation of Cr and MPs. The sorption experiment suggested that the photoreduction of Cr(VI) partly took place on the MPs surface. Based on our findings, we further speculated that ROS generated from MPs-DOM may also play a role. In the natural environment, solar irradiation may draw similar conclusion but the phototransformation process may be quite slow. Also, many environment factors, such as natural DOM and Fe(II), may interfere this procedure [17], which merits further investigations.

### 3.3 Biolability of MPs-DOM and its environmental filtration to microbial communities

**Fig. 4:**
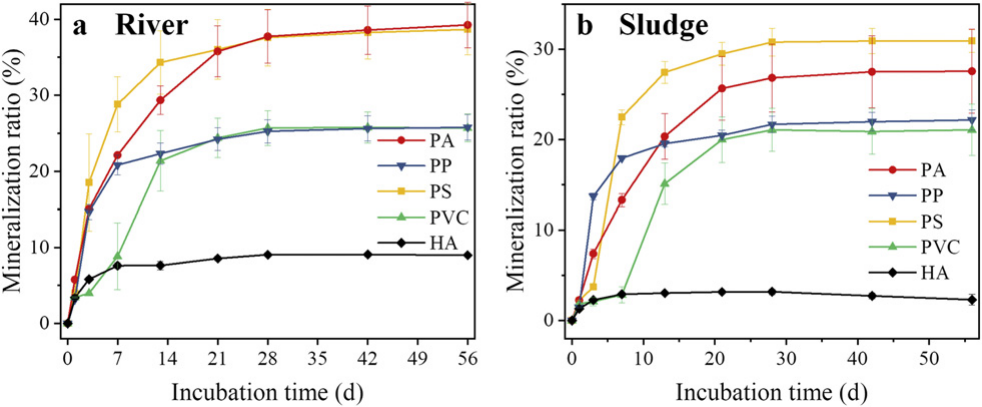
**Dynamics of MPs-DOM biodegradation during 56-day incubation at 25 °C using riverine (a) or sludge inocula (b) based on cumulative CO_2_ emissions**.

The photochemical experiment suggested the potentially significant role of MPs-DOM in the phototransformation of coexisting pollutants (e.g., Cr in this study), which attracted our attention on the ecological effects (e.g., greenhouse gas emission) of MPs-DOM itself. The biodegradability of MPs-DOM varied with MPs type and inoculum source (Fig. 4, Table 1). After 56 days of incubation, the mineralization ratio of MPs-DOM followed a descending order of PS ≥ PA >PP ≥ PVC > SRHA (Fig. 4). As indicated by the two-pool exponential model, MPs-DOM contained a higher proportion of labile DOM than SRHA (Table 1). The amount of labile fraction in MPs-DOM followed the sequence of PS > PA > PVC >PP. The half-life of the labile fractions in MPs-DOM had the order of PVC > PA > PS > PP, while that of the recalcitrant fractions had the order of PVC > PP > PS > PA. Typically, PVC-DOM contained a relatively small proportion of the labile fraction, possessed the longest half-life with respect to both its labile and recalcitrant fractions, and thus showed the weakest biodegradability. Although containing less labile fractions, PP-DOM exhibited a much shorter half-life and thus showed a higher mineralization ratio than PVC-DOM. PS and PA contained a higher proportion of labile DOM, possessed a shorter half-life, and therefore exhibited a higher mineralization ratio. Interestingly, the MPs-DOM inoculated with sludge microbes had a lower mineralization ratio than the MPs-DOM inoculated with riverine microbes. The inoculum sources also affected the fitting results of the two-pool exponential model. This indicated that different microbes preferred to utilize different carbon sources, which led to the difference in the final degradation ratio. The photoproduced MPs-DOM at 40 h mainly comprised long-chain alkanes, high-molecular-weight acids, and benzoic ether (Table S2), which were related to monomers and oligomers that initially formed the polymer chain [48]. After 56 days of incubation, those substances were further degraded, among which PVC-DOM showed the weakest degradation.

**Table 1:** **Quantitative measures of the MPs-DOMs biodegradation after 56 days incubation based on cumulative CO_2_ emission**.

Sample	Labile DOM (%)	Stable DOM (%)	*k*_1_ (d^–1^)	*k*_2_ (× 10^–3^ d^–1^)	Hafe-life 1 (d)	Half-life 2 (y)	*r*^2^
PA-R	34.74 ± 5.83a	65.26 ± 5.83e	0.15 ± 0.04bc	1.42 ± 2.1a	4.61	1.34	0.971
PP-R	23.35 ± 2.32cd	76.65 ± 2.32bc	0.28 ± 0.07bc	0.64 ± 0.83bcd	2.51	2.99	0.968
PS-R	35.99 ± 6.06a	64.01 ± 6.06e	0.21 ± 0.08bc	0.79 ± 2.5abc	3.23	2.40	0.935
PVC-R	26.80 ± 2.68bc	73.20 ± 2.68cd	0.09 ± 0.03c	0.10 ± 0.00cd	7.80	18.99	0.919
SRHA-R	7.70 ± 0.58e	92.30 ± 0.58a	0.50 ± 0.12ab	0.32 ± 0.18bcd	1.37	5.95	0.977
PA-S	27.58 ± 1.00bc	72.42 ± 1.00cd	0.11 ± 0.01bc	1.00 ± 0.00ab	6.53	1.90	0.993
PP-S	20.15 ± 2.29d	79.85 ± 2.29b	0.28 ± 0.08bc	0.49 ± 0.79bcd	2.44	3.86	0.961
PS-S	29.70 ± 2.16ab	68.35 ± 2.16de	0.14 ± 0.04bc	0.70 ± 0.00abcd	5.39	2.71	0.948
PVC-S	22.82 ± 3.60cd	77.18 ± 3.60bc	0.07 ±0.03c	0.10 ± 0.00cd	10.62	18.99	0.894
SRHA-S	2.70 ± 0.47e	97.30 ± 0.47a	0.86 ± 0.59a	0.01 ± 0.15d	1.23	138.01	0.822

Note: R and S represented the riverine and sludge microbe inoculums, respectively. The R and S inoculated MPs-DOMs were abbreviated as XX-R and XX-S, where XX referred to the MPs-DOMs types or SRHA. For example, PA-R can be interpreted as riverine microbes inoculated PA-DOM. *k*_1_ and *k*_2_ represent the biodegradation rate constant for labile and recalcitrant carbon, respectively. Half-life 1 and Half-life 2 refer to the half-life of the labile and recalcitrant DOM pools, respectively. Different lowercase letters represent significant differences between different treatments (*p* < 0.05).

**Fig. 5:**
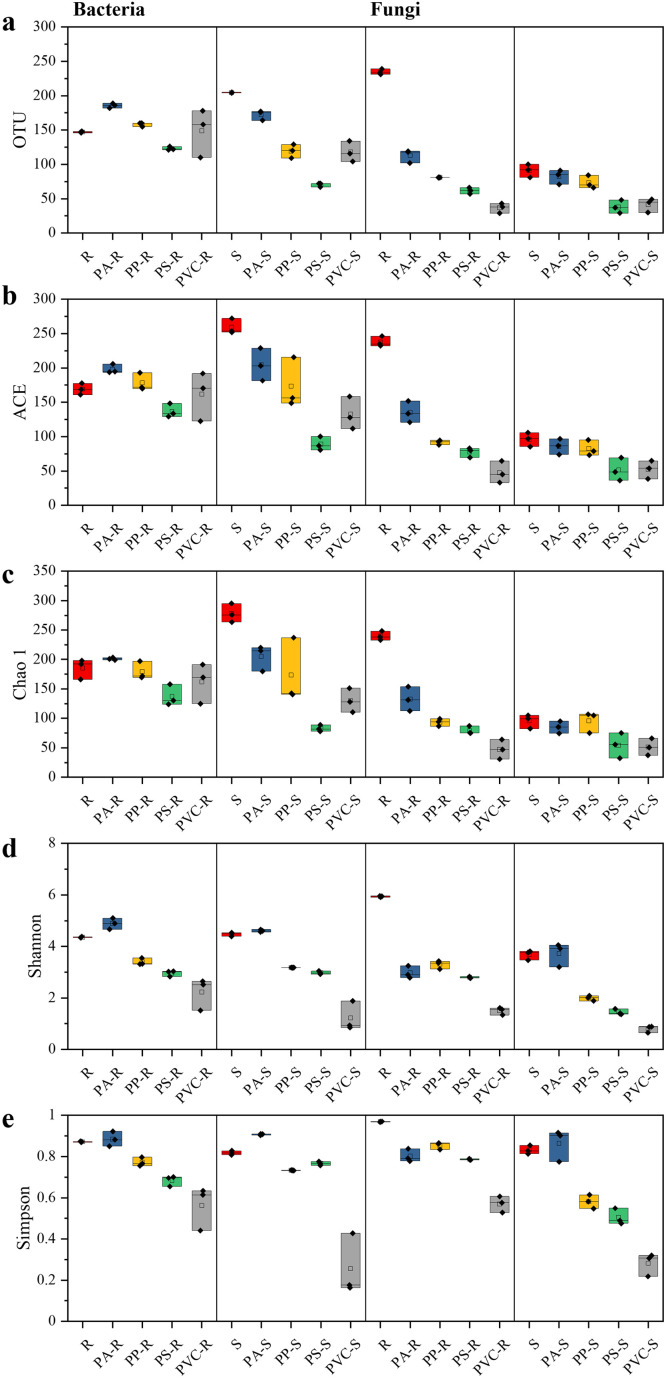
**Richness (a‒c) and diversity (d‒e) indices of bacterial and fungal species in MPs-DOMs and corresponding Riverine (R) and Sludge (S) inocula**.

Different MPs-DOMs contain various available carbon sources and can thus affect microbial succession [16]. Compared with the original inocula, the abundance and diversity of bacteria and fungi were greatly modified by different MPs-DOM sources (Fig. 5, Table S3). With respect to samples with the riverine inoculum, MPs-DOM lowered the ACE, Chao 1, Shannon, and Simpson indices for both the bacterial 16S gene and the fungal ITS gene, except for those of PA-DOM and the ACE index of PP-DOM for the 16S gene. For samples with the sludge inoculum, MPs-DOM also lowered this index except for the Shannon and Simpson index of PA-DOM for both the bacterial 16S gene and the fungal ITS gene. As such, the abundance and diversity of bacteria and fungi generally decreased after 56 days of incubation with different MPs-DOM sources. Overall, PA-DOM showed the highest ACE, Chao 1, Shannon, and Simpson values, while PVC-DOM had the lowest values. Additionally, MPs-DOM had stronger filtration effects on the fungal genes for the riverine inoculum and the bacterial genes for the sludge inoculum. Such results suggested that different MPs-DOM sources possessed varying degrees of filtration to the microbial communities, and the filtration effects were partly dependent on the inoculum sources.

**Fig. 6:**
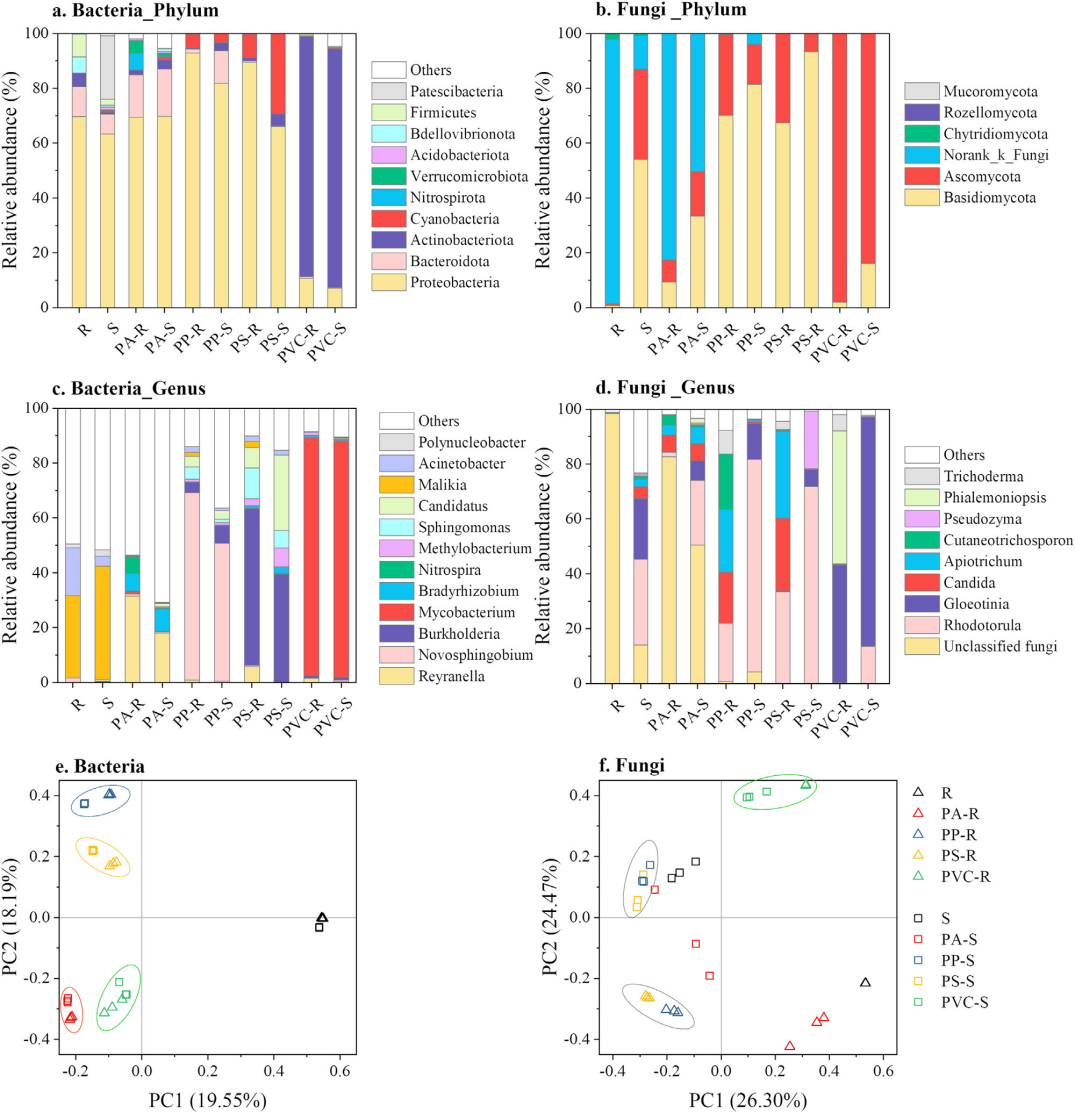
(a-d) Mean relative abundance of bacteria (a and c) and fungi (b and d) at the phylum (a-b) and genus levels (c-d) in the original inocula and corresponding inoculated MPs-DOM. (e-f) Principal coordinate analysis (PCoA) of bacterial (e) and fungal (f) communities in the original inocula and corresponding inoculated MPs-DOM using the weighted Fast UniFrac metric. The values in parentheses at the x- (the first coordinate, PC1) and y-axes (the second coordinate, PC2) represent the percentages of the community variation interpreted. The PC1 to PC3 (the third coordinate) plotted PCoA analysis is provided in Fig. S6. Here, R and S represent the riverine and sludge microbe inocula, respectively. The R- and S-inoculated MPs-DOM are abbreviated XX-R and XX-S, where XX refers to the MPs-DOM types. For example, PA-R can be interpreted as riverine microbes inoculated with PA-DOM.

In addition to the shifts in microbial abundance and diversity, MPs-DOMs also changed the community composition (Fig. 6). For bacteria, the riverine inoculum mainly comprised Proteobacteria (69.7%) > Bacteroidota (10.9%) > Firmicutes (8.3%) > Bdellovibrionota (6.0%) > Actinobacteriota (4.9%), while the sludge inoculum contained Proteobacteria (63.3%) > Patescibacteria (23.1%) > Bacteroidota (7.2%) > Firmicutes (2.2%) at the phylum level (Fig. 6a). After 56 days of incubation, the bacterial compositions of the same MPs-DOM sources were similar despite the different inoculum types. PA-DOM mainly comprised Proteobacteria (69.4%–69.8%) > Bacteroidota (15.4%–17.2%) and possessed increasing proportions of Nitrospirota and Verrucomicrobiota compared to the original inocula (*p* < 0.01), which was supported by the good performance of PA as nitrifying bacteria support [49] and the enriched Verrucomicrobiota by biodegradable microplastics application [50]. In addition, PA-DOM inoculated by riverine microbes possessed a higher proportion of Nitrospirota and Verrucomicrobiota (*p* < 0.01), suggesting the important role of original inocula in microbial succession. PP-DOM was mainly composed of Proteobacteria (81.7%–92.8%), Bacteroidota (1.5%–11.9%), Cyanobacteria (3.1%–5.1%), and Actinobacteriota (0.4%–3.0%), while PS-DOM had the order of Proteobacteria (66.0%–89.4%) > Cyanobacteria (8.6%–29.3%) > Actinobacteriota (1.2%–4.3%). The enrichment of Cyanobacteria was potentially attributed to its utilization of PP-DOM and PS-DOM carbon [51,52]. In addition, PVC-DOM mainly comprised Actinobacteriota (87.1%–87.5%) and Proteobacteria (7.2%–10.7%), with the former having a higher tolerance to PVC-DOM [53]. Proteobacteria, which included many nitrogen-fixing bacteria [54], was the predominant phylum in both the original inoculum and the MPs-DOM samples, except for PVC-DOM, which can be explained by the weak tolerance of Proteobacteria to PVC particles [53]. The results suggested that PVC-DOM had the strongest filtration to the bacterial composition, which was consistent with the shifts in bacterial abundance and diversity (Figs. 5,6a, and Table S3). At the genus level, the predominant genera were *Reyranella, Novosphingobium, Burkholderia*, and *Mycobacterium* for PA-DOM, PP-DOM, PS-DOM, and PVC-DOM, respectively (Fig. 6c). Typically, PA-DOM also significantly enriched the abundance of *Nitrospira* and *Bradyrhizobium*, with the former playing a pivotal role in nitrification as an aerobic chemolithoautotrophic nitrite-oxidizing bacterium [55] and the latter as a nitrogen-fixing symbiotic bacterium [56]. In addition, the profoundly enriched *Novosphingobium* in PP-DOM was associated with the biodegradation of polycyclic aromatic hydrocarbons [57,58], which was confirmed by the most profoundly lowered benzene-containing molecules according to GC–MS (Table S2). Moreover, Mycobacterium is acid fast and resistant to chlorine [59] and therefore gradually evolved into the dominant bacterial group in PVC-DOM. As such, different MPs-DOMs favored the growth of various bacteria. With respect to the cluster of orthologous groups (COG) function predicted by PICRUSt (Fig. S5a), PP-DOM and PVC-DOM demonstrated stronger lipid transport and metabolism but weaker amino and carbohydrate transport and metabolism, which served as an explanation for their low mineralization ratio. In addition, the Kyoto Encyclopedia of Genes and Genomes (KEGG) function prediction suggested that PVC-DOM bacteria were more involved in metabolism but less involved in cellular and environmental/genetic information processes (Fig. S5b).

Regarding fungi, the mean abundance of fungal phyla followed a descending order of Norank_k_Fungi (96.5%) > Chytridiomycota (2.1%) > Basidiomycota (0.7%) > Ascomycota (0.6%) for riverine fungi and Basidiomycota (54.1%) > Ascomycota (32.9%) > Norank_k_Fungi (12.2%) > Chytridiomycota (0.6%) for sludge fungi (Fig. 6b). After 56 days of incubation, the fungal compositions of PS-DOM and PP-DOM mainly comprised Basidiomycota (67.5%–93.2%) > Ascomycota (6.8%–32.5%), while PVC-DOM possessed a higher proportion of Ascomycota (83.8%–98.0%). Notably, PA-DOM contained a rather high proportion of Norank_k_Fungi, which added to the uncertainty in the description of corresponding fungal succession. At the genus level, MPs-DOMs inoculated with the sludge microbes possessed higher proportions of *Rhodotorula* and *Gloeotinia* than those inoculated the riverine microbes (Fig. 6d), which was potentially attributed to the varied inoculum sources.

The shifts in microbial communities in various MPs-DOM sources were also displayed by PCoA based on the weighted fast UniFrac metric (Figs. 6e, f, and S6). Principal coordinate 1 (PC1), principal coordinate 2 (PC2) and principal coordinate 3 (PC3) explained more than 50% and clearly demonstrated the succession of bacterial and fungal communities by MPs-DOM sources. PC1 separated the bacterial communities of the original inoculum from those of MPs-DOM, PC2 separated the bacterial communities of PA-DOM and PVC-DOM from those of PP-DOM and PS-DOM, and PC3 separated those of PA-DOM and PS-DOM from those of PP-DOM and PVC-DOM (Figs. 6e, S6a). The differences in the same MPs-DOM source with different inocula were much smaller than those among different MPs-DOM sources. With respect to fungi, the riverine inoculum was completely separated from the sludge inoculum by PC1 and PC2 (Fig. 6f). PC2 generally separated MPs-DOMs inoculated with the riverine microbes from those inoculated with the sludge inoculated, except for PA-S and PVC-R. In addition, PC1 and PC2 also separated the fungal communities of PVC-DOM from those of other MPs-DOM sources. Such results further supported the conclusion that bacterial and fungal communities were altered by different MPs-DOM sources, with a stronger filtration effect being observed for PVC-DOM in both bacterial and fungal communities.

Overall, MPs-DOMs had higher degradability than SRHA and made significant contributions to greenhouse gas emissions. Also, the microbial compositions were significantly altered by different MPs-DOM sources. Moreover, as indicated by previous records, the microbial compositions in MPs-DOM may varied with incubation time [60]. Thus, it could be expected that MPs-DOMs had filtration effects on the microbial composition and function, and the microbial communities had continuous and dynamic effects on the mineralization of the DOC in MPs-DOMs.

## 4 Conclusion

Plastics are still widely used for many years to come due to their current irreplaceability. To date, there is a dearth of information on the effects of MPs and MPs-DOM on the transformation of coexisting pollutants and corresponding ecological effects once they enter the aquatic environment. This study demonstrated that the photooxidation of both MPs and Cr(III) was driven by ROS. MPs addition inhibited the photooxidation of Cr(III) and induced the photoreduction of Cr(VI), potentially by surface adsorption. During this process, MPs were degraded and Cr(VI) was transformed into less hazardous Cr(III), which is a win-win solution for the treatment of MPs and co-existing contaminants (e.g. Cr(VI) in this study). In addition, large amounts of MPs-DOM were generated during the photoirradiation process, and comparable amounts of ROS were produced solely by MPs and MPs-DOM solutions at different irradiation times. These ROS could affect the further photodegradation of MPs and MPs-DOM and may also have an impact on the phototransformation of Cr. Compared to riverine humic acid, MPs-DOM exhibited higher bioavailability, suggesting the potential effects of MPs-DOM on the carbon cycle. The mineralization ratio of the four experimental MPs-DOMs followed a descending order of PS ≥ PA >PP ≥ PVC. Upon entering the aquatic environment, MPs-DOM also possessed strong filtration effects on the bacterial and fungal communities. Bacterial succession was highly related to MPs-DOM sources, while fungal succession was affected by both the MPs-DOM source and the original inoculum type. Typically, PVC-DOM had the strongest filtration effects on the bacterial and fungal communities and the corresponding functional gene expression. This study proposed a win–win solution for Cr(VI) photoreduction and MPs degradation, highlighted the potential significant role of MPs-DOMs in the phototransformation of coexisting pollutants, greenhouse emissions, and microbial succession in aquatic environments and called for more attention to be paid to the ecological effects of MPs-DOM. Based on the phototransformation experiment and short-term laboratory incubation experiment, we confirmed the significant role of MPs-DOM in the transformation of coexisting pollutants, as well as greenhouse gas emissions and microbial succession in aquatic environments. The results expanded our understanding of the fate and ecological effects of MPs and coexisting pollutants and highlighted the importance of MPs-DOM interactions. It is worth noting that nanoplastics are very likely to exist in the MPs-DOM solution. Further investigation should be conducted regarding the effects of nanoplastics.

## Declaration of competing interest

The authors declare that they have no conflicts of interest in this work.

## Appendix Supplementary materials

Supporting information: The supporting information contains six figures and three tables and presents detailed results about the photodegradation of MPs, UV-vis and EEM spectra of four MPs-DOMs, ROS generated by MPs and MPs-DOM, gene prediction of bacteria in MPs-DOMs, PCoA analysis of bacteria and fungi, the elemental composition of MPs, the detected molecules in MPs-DOMs by GC–MS, and the richness and diversity indices of microbial community in MPs-DOMs. Image, application 1
