# Supplementary material for: The photo-redox of chromium regulated by microplastics (MPs) and MPs-derived dissolved organic matter (MPs-DOM) and the CO2 emission of MPs-DOM
Source: Fundam Res. 2022 Aug 24;4(6):1576–85. doi: 10.1016/j.fmre.2022.08.009 (PMC11670657; doi:10.1016/j.fmre.2022.08.009)
Supplement: Supplementary file 1 — Supporting information: The supporting information contains six figures and three tables and presents detailed results about the photodegradation of MPs, UV-vis and EEM spectra of four MPs-DOMs, ROS generated by MPs and MPs-DOM, gene prediction of bacteria in MPs-DOMs, PCoA analysis of bacteria and fungi, the elemental composition of MPs, the detected molecules in MPs-DOMs by GC–MS, and the richness and diversity indices of microbial community in MPs-DOMs. [file mmc1.docx]

**Supporting Information**

**The photo-redox of chromium regulated by microplastics (MPs) and MPs-derived dissolved organic matter (MPs-DOM) and the CO2 emission of MPs-DOM**

Enyao Zhang^a,1^, Yalan Chen^a,1^, Yang Li^a^, Ke Sun^a,^*, Yan Yang^a^, Bo Gao^b^, Baoshan Xing^c^

*^a^ State Key Laboratory of Water Environment Simulation, School of Environment, Beijing Normal University, Beijing 100875, China*

*^b^* *State Key Laboratory of Simulation and Regulation of Water Cycle in River Basin, China Institute of Water Resources and Hydropower Research, Beijing 100038, China*

*^c^ Stockbridge School of Agriculture, University of Massachusetts, Amherst, MA 01003, USA*

*Corresponding author: (86)‐10‐58807493 (phone), (86)‐10‐58807493 (fax), email: sunke@bnu.edu.cn (K. SUN).

^1^ These authors contributed equally to this work.

**Supplementary Information**

Number of pages: 7

Number of Figures: 6

Number of Tables: 1

# Supporting Figures

**
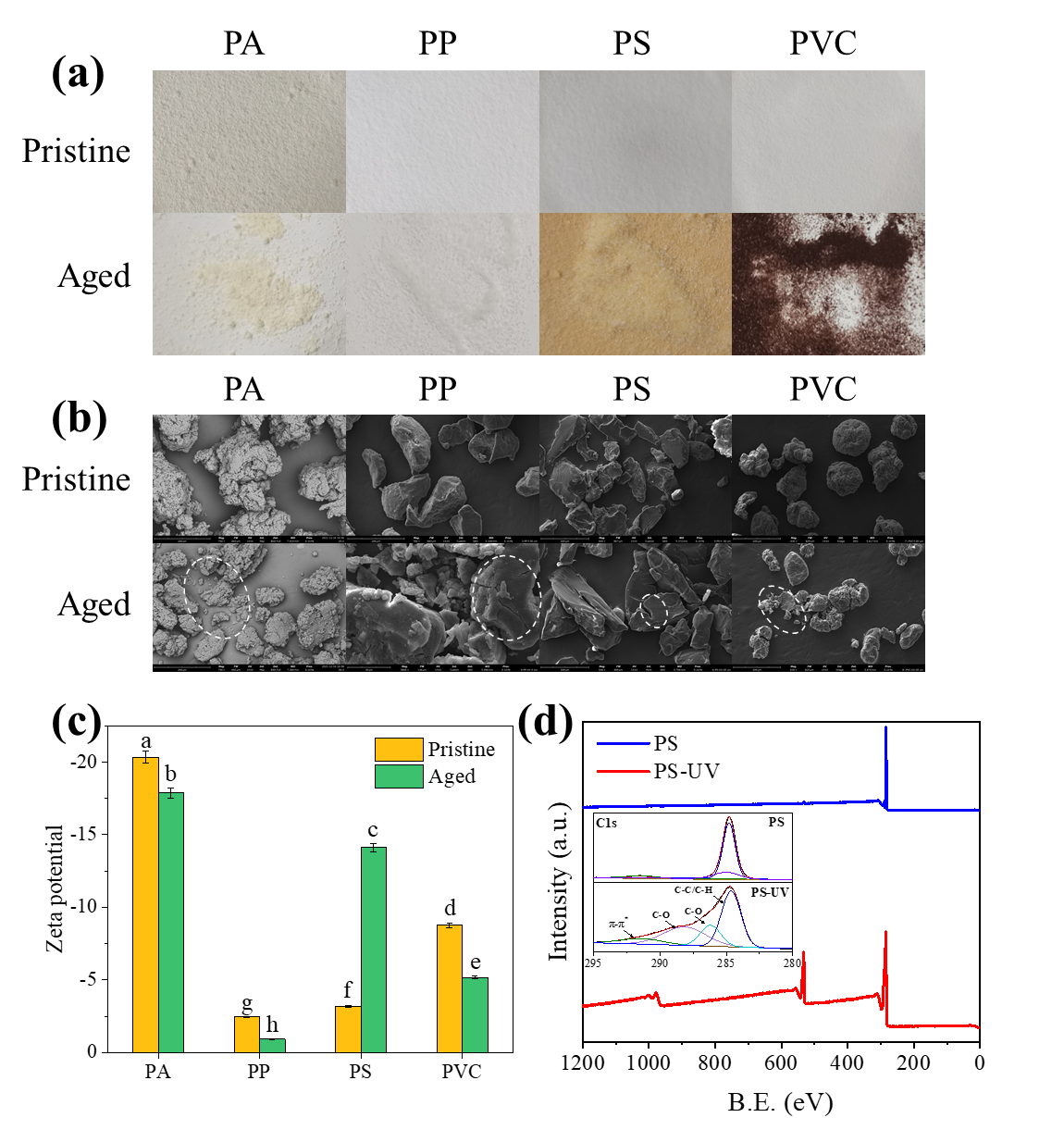
Fig. S1** The color (a), morphology (b), surface potential (c), and surface functional groups (d) changes of four experimental MPs after 8 h UV irradiation by camera, SEM, zeta potential analyzer, and XPS. Different lowercase letters represent significant differences between different treatments (*p* < 0.05).


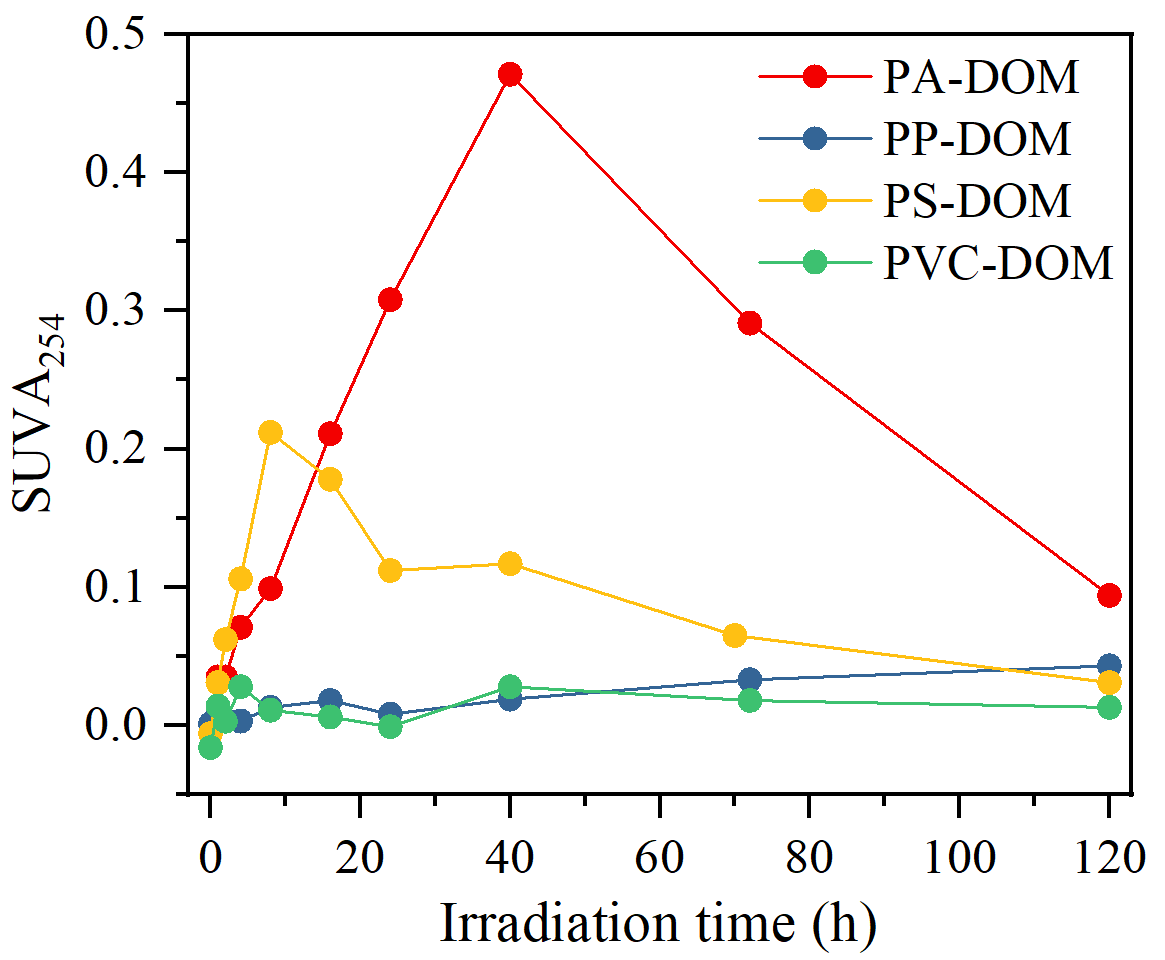


**Fig. S2** Changes of SUVA_254_ values of MPs-DOM at different irradiation moments.


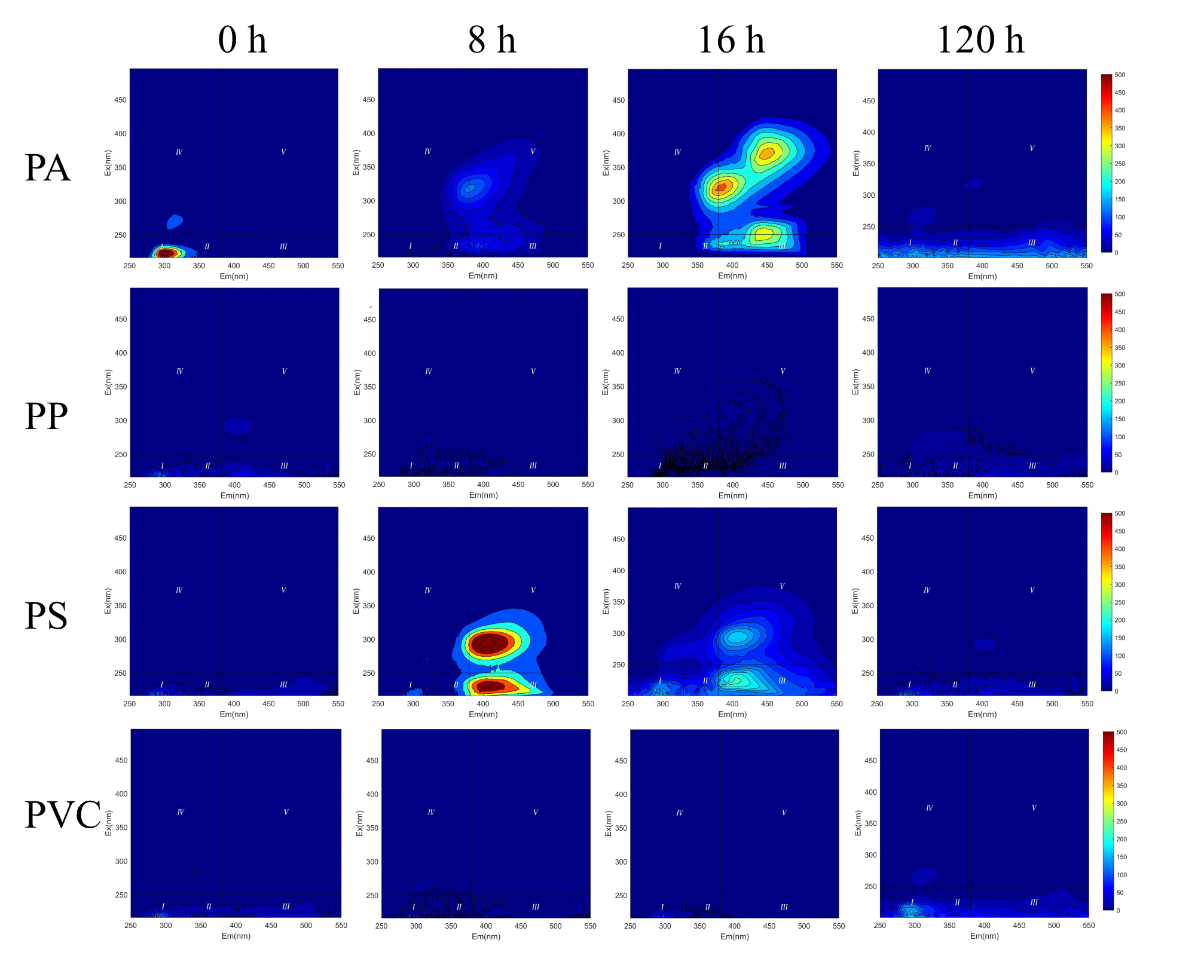


**Fig. S3** Changes of EEM spectra of PA-DOM, PS-DOM, PP-DOM, and PVC-DOM after 0, 8, 16, and 120 h irradiation time.


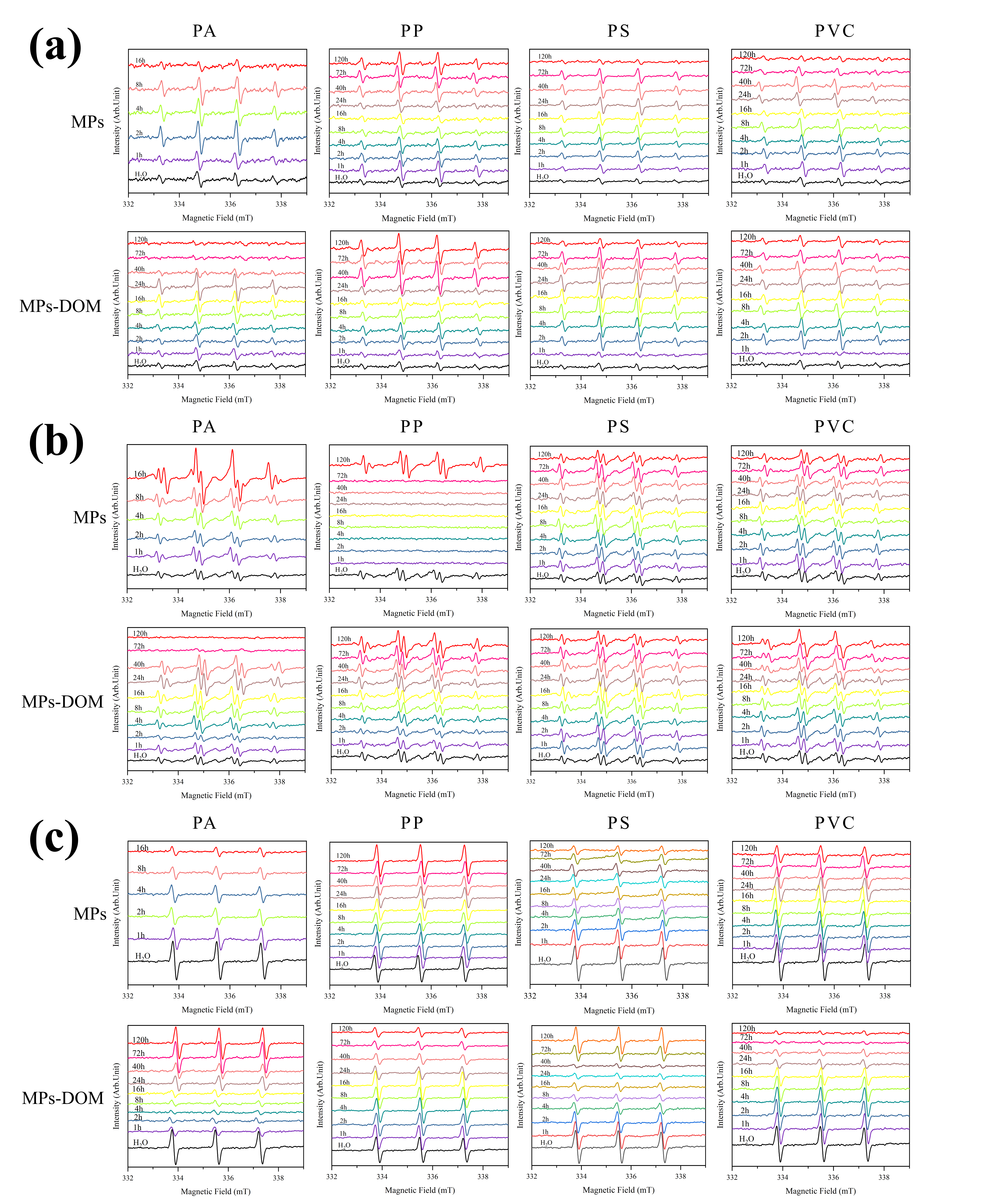


**Fig. S4** Intensity changes of DMPO (a), BMPO (b), and TEMP spin adduct (c) generated by MPs and corresponding MPs-DOMs at different irradiation moments according to semiquantitative ESR spectra.


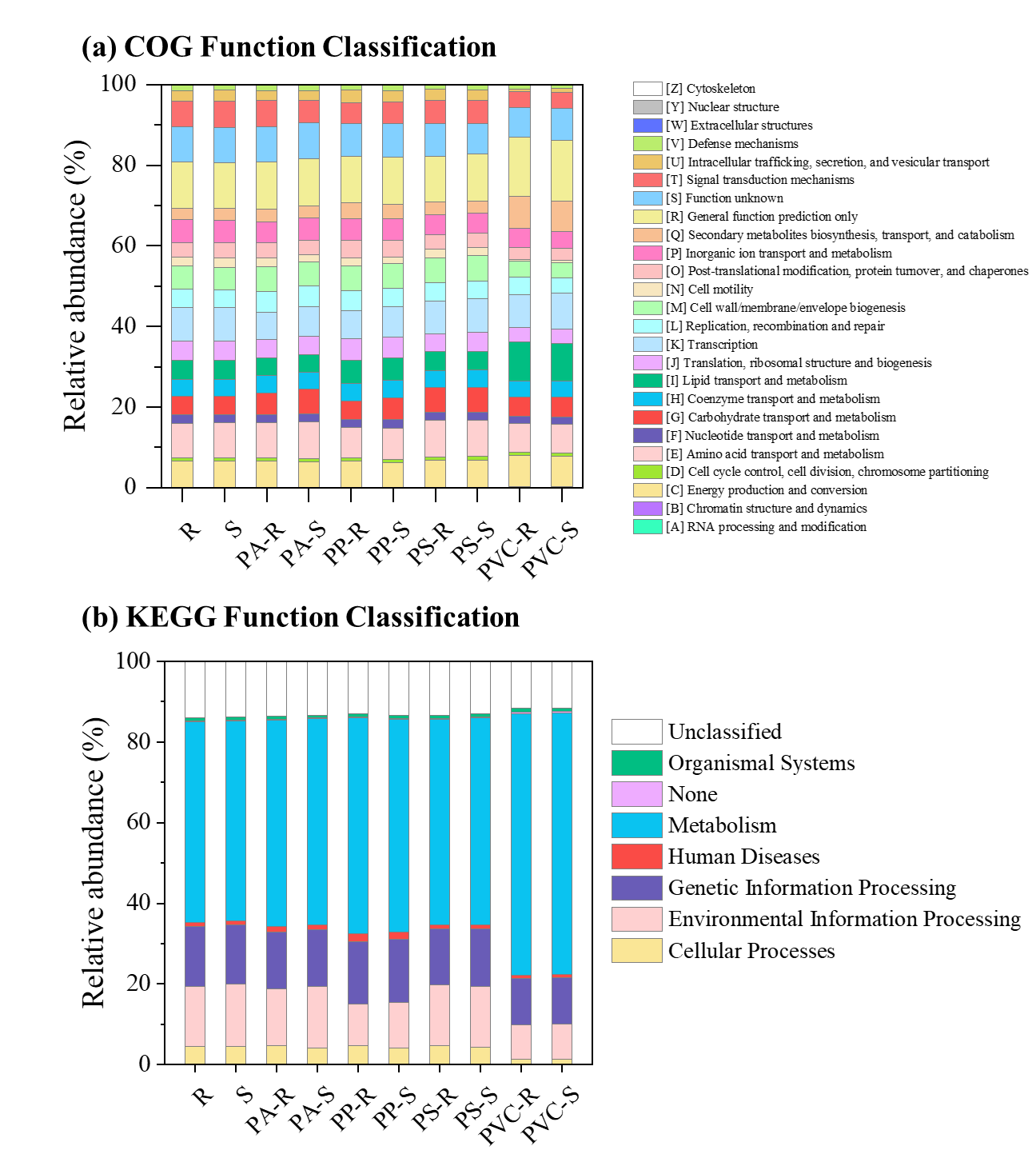


**Fig. S5** Mean relative abundance of predicted COG (a) and KEGG orthologs at level 1 (b) by PICRUSt. Here, R and S represented the riverine and sludge microbe inoculums, respectively. The R and S inoculated MPs-DOMs were abbreviated as XX-R and XX-S, where XX referred to the MPs-DOMs types. For example, PA-R can be interpreted as riverine microbes inoculated PA-DOM.


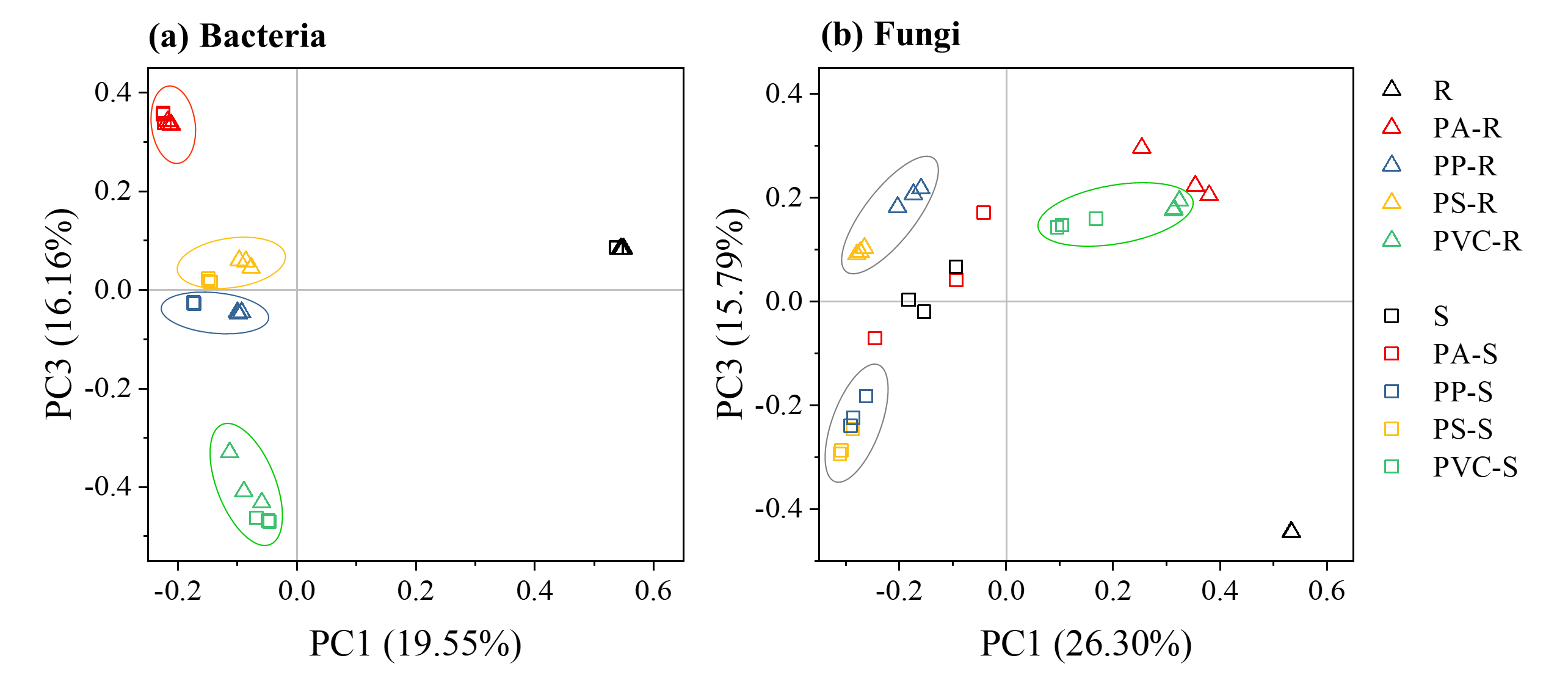


**Fig. S6** Principal coordinate analysis (PCoA) analysis of bacteria (a) and fungi (b) communities in the original inoculums and corresponding inoculated MPs-DOMs using the weighted Fast UniFrac metric. The values in parentheses at x- (the first coordinate, PC1) and y-axes (the third coordinate, PC3) represented the percentages of the community variation interpreted. Here, R and S represented the riverine and sludge microbe inoculums, respectively. The R and S inoculated MPs-DOMs were abbreviated as XX-R and XX-S, where XX referred to the MPs-DOMs types. For example, PA-R can be interpreted as riverine microbes inoculated PA-DOM.

**Table S1** Elemental composition (C, H, O, and N) of four experimental MPs.

| **Sample** | **C** | **H** | **N** | **O** | **H/C** | **O/C** |
| --- | --- | --- | --- | --- | --- | --- |
| PP | 85.4±0.3 | 14.2±0.1 | ‒ | ‒ | 0.17±0.00 | ‒ |
| PS | 91.8±0.5 | 7.8±0.2 | ‒ | ‒ | 0.08±0.00 | ‒ |
| PVC | 38.7±0.7 | 4.7±0.1 | ‒ | ‒ | 0.12±0.01 | ‒ |
| PA | 63.5±0.4 | 10.1±0.6 | 12.3±0.1 | 14.1±0.8 | 0.16±0.01 | 0.22±0.01 |

**Table S2** Assigned formulas and peak area of the detected molecules in MPs-DOMs pre- and post-incubation.

| Time (min) | Molecular formula | Structural formula | Peak area | Sample |
| --- | --- | --- | --- | --- |
| 8.411 | C_9_H_16_O_2_ | 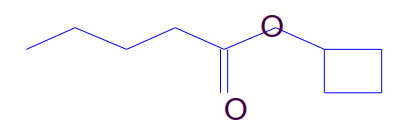 | 1.39E+04 | PA |
|  |  |  | – | PA-56 |
|  |  |  | 2.39E+07 | PP |
|  |  |  | – | PP-56 |
|  |  |  | – | PS |
|  |  |  | – | PS-56 |
|  |  |  | – | PVC |
|  |  |  | – | PVC-56 |
| 8.532 | C_12_H_24_O_2_ | 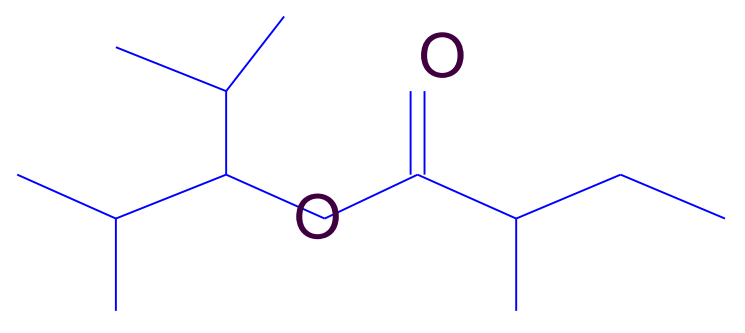 | 1.39E+04 | PA |
|  |  |  | – | PA-56 |
|  |  |  | 3.14E+07 | PP |
|  |  |  | – | PP-56 |
|  |  |  | – | PS |
|  |  |  | – | PS-56 |
|  |  |  | – | PVC |
|  |  |  | – | PVC-56 |
| 9.338 | C_6_H_4_O_2_ | 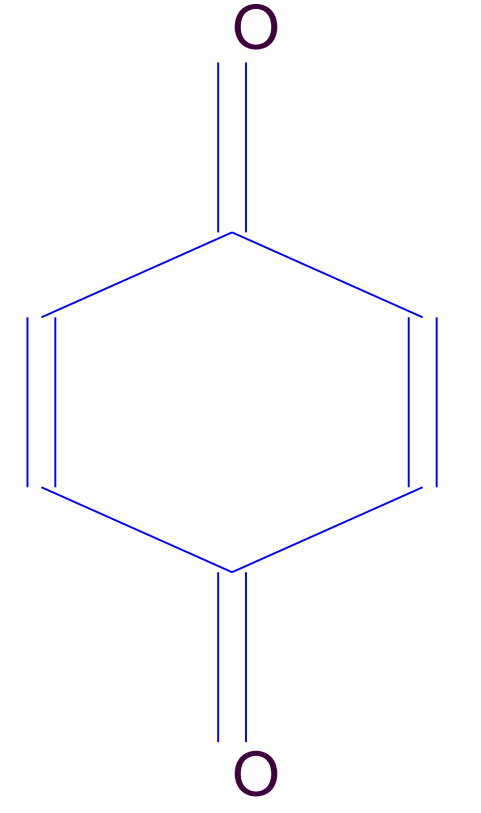 | 5.00E+05 | PA |
|  |  |  | – | PA-56 |
|  |  |  | – | PP |
|  |  |  | – | PP-56 |
|  |  |  | – | PS |
|  |  |  | – | PS-56 |
|  |  |  | – | PVC |
|  |  |  | – | PVC-56 |
| 10.820 | C_29_H_60_ | 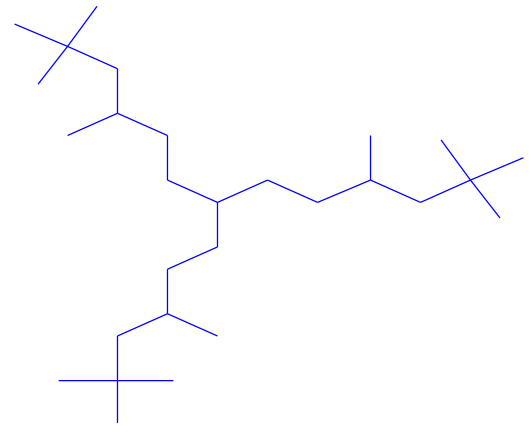 | 9.93E+07 | PA |
|  |  |  | 2.24E+07 | PA-56 |
|  |  |  | 3.00E+08 | PP |
|  |  |  | 4.14E+04 | PP-56 |
|  |  |  | 8.60E+07 | PS |
|  |  |  | 1.79E+07 | PS-56 |
|  |  |  | 1.22E+07 | PVC |
|  |  |  | 8.07E+06 | PVC-56 |
| 12.905 | C_7_H_8_ON_2_ | 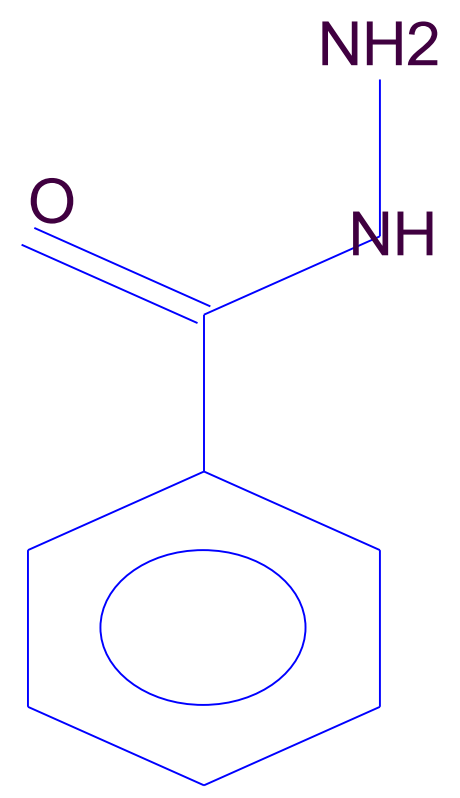 | 5.92E+07 | PA |
|  |  |  | 6.07E+06 | PA-56 |
|  |  |  | 1.58E+08 | PP |
|  |  |  | 1.21E+04 | PP-56 |
|  |  |  | 3.56E+07 | PS |
|  |  |  | 4.72E+06 | PS-56 |
|  |  |  | – | PVC |
|  |  |  | – | PVC-56 |
| 14.188 | C_17_H_30_ | 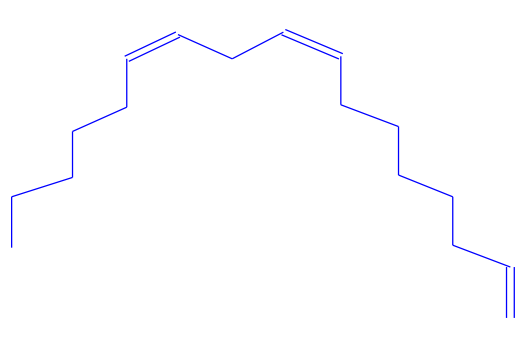 | 1.60E+07 | PA |
|  |  |  | 1.48E+06 | PA-56 |
|  |  |  | 3.59E+07 | PP |
|  |  |  | 1.20E+06 | PP-56 |
|  |  |  | 8.22E+06 | PS |
|  |  |  | 1.34E+06 | PS-56 |
|  |  |  | – | PVC |
|  |  |  | – | PVC-56 |
| 16.848 | C_21_H_34_O_4_ | 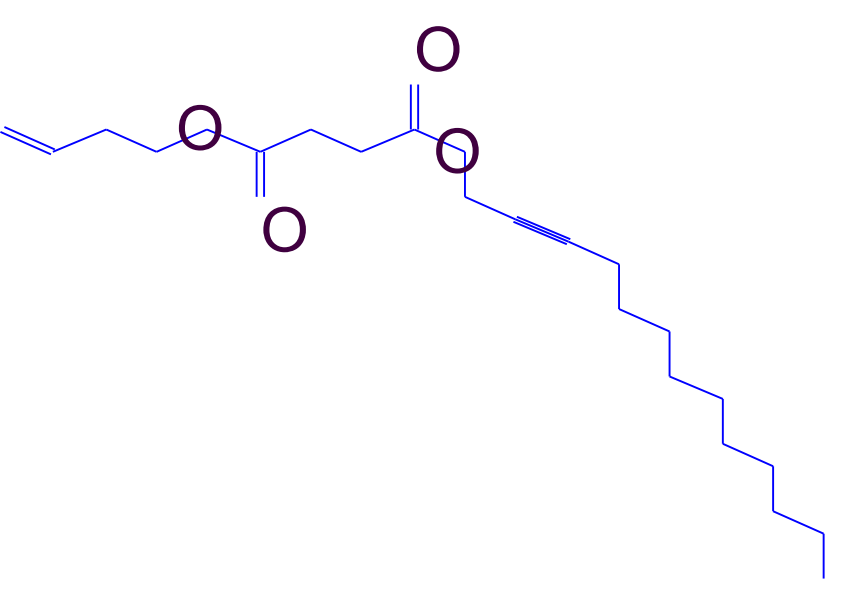 | 4.74E+06 | PA |
|  |  |  | – | PA-56 |
|  |  |  | 1.35E+07 | PP |
|  |  |  | – | PP-56 |
|  |  |  | 2.33E+06 | PS |
|  |  |  | – | PS-56 |
|  |  |  | – | PVC |
|  |  |  | – | PVC-56 |
| 17.537 | C_18_H_30_O_2_ | 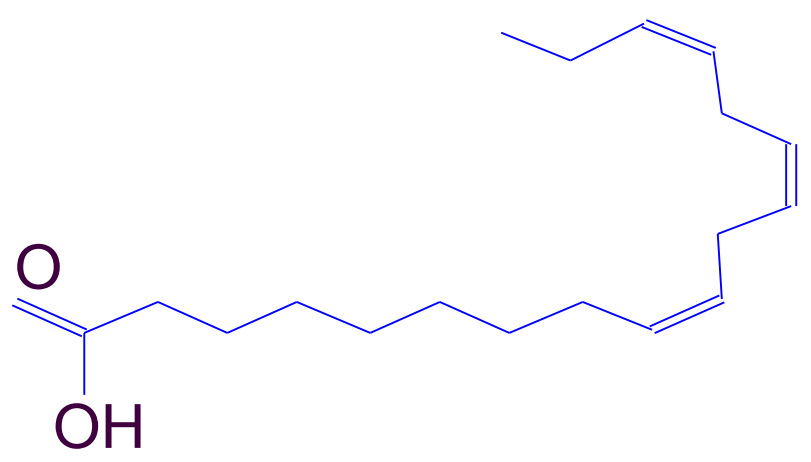 | 4.25E+11 | PA |
|  |  |  | 6.91E+09 | PA-56 |
|  |  |  | 1.30E+09 | PP |
|  |  |  | 9.30E+06 | PP-56 |
|  |  |  | 1.67E+08 | PS |
|  |  |  | 1.43E+07 | PS-56 |
|  |  |  | 3.88E+06 | PVC |
|  |  |  | 2.36E+06 | PVC-56 |
| 17.618 | C_10_H_16_ | 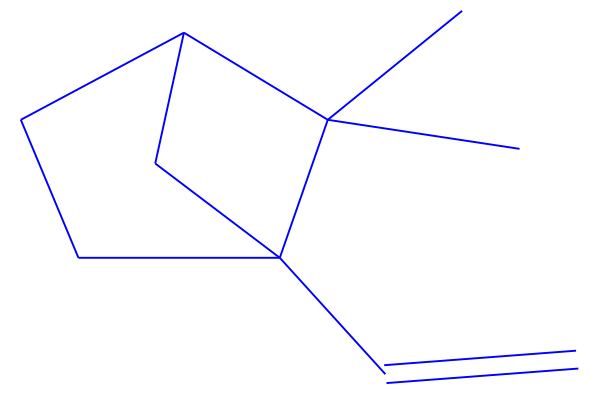 | – | PA |
|  |  |  | – | PA-56 |
|  |  |  | 5.06E+6 | PP |
|  |  |  | – | PP-56 |
|  |  |  | – | PS |
|  |  |  | – | PS-56 |
|  |  |  | – | PVC |
|  |  |  | – | PVC-56 |
| 18.075 | C_11_H_16_O_2_ | 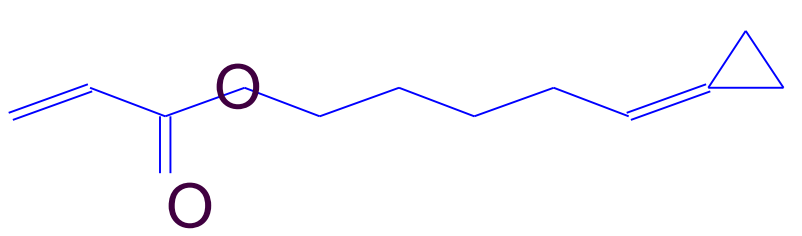 | 1.45E+06 | PA |
|  |  |  | – | PA-56 |
|  |  |  | 1.35E+07 | PP |
|  |  |  | – | PP-56 |
|  |  |  | 2.33E+06 | PS |
|  |  |  | – | PS-56 |
|  |  |  | – | PVC |
|  |  |  | – | PVC-56 |
| 18.877 | C_21_H_35_O | 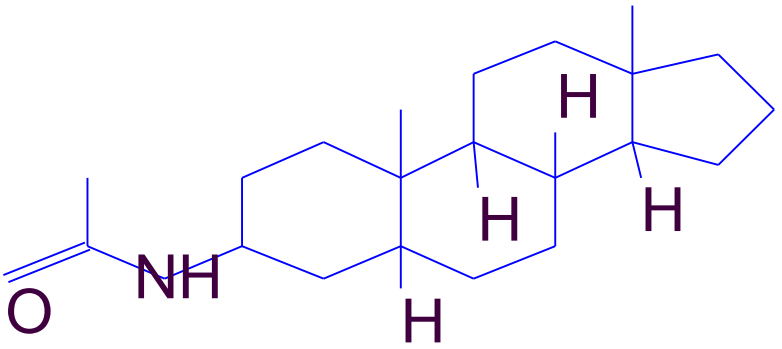 | 1.61E+06 | PA |
|  |  |  | – | PA-56 |
|  |  |  | 4.59E+08 | PP |
|  |  |  | – | PP-56 |
|  |  |  | 8.77E+05 | PS |
|  |  |  | – | PS-56 |
|  |  |  | – | PVC |
|  |  |  | – | PVC-56 |
| 23.831 | C_9_H_10_O_3_ | 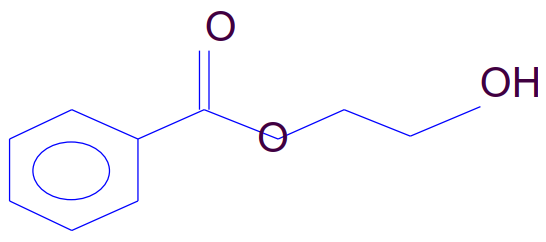 | 5.76E+07 | PA |
|  |  |  | 5.95E+05 | PA-56 |
|  |  |  | 1.44E+08 | PP |
|  |  |  | – | PP-56 |
|  |  |  | 1.78E+07 | PS |
|  |  |  | 8.89E+05 | PS-56 |
|  |  |  | 3.97E+06 | PVC |
|  |  |  | 4.50E+05 | PVC-56 |

**Table S3** Richness and diversity indices of bacterial and fungal species in MPs-DOMs and corresponding Riverine (R) and Sludge (S) inoculums.

| Sample | Richness | | |  | Diversity | |
| --- | --- | --- | --- | --- | --- | --- |
|  | OUT | ACE | Chao 1 |  | Shannon | Simpson |
| ***Bacteria*** | | | | | | |
| R | 147.00 ± 1.00cd | 169.09 ± 8.25bcde | 185.44 ± 16.98bc |  | 4.36 ± 0.02a | 0.87 ± 0.00ab |
| PA-R | 185.67 ± 3.51ab | 198.10 ± 6.66bc | 201.15 ± 1.83b |  | 4.89 ± 0.22a | 0.88 ± 0.04ab |
| PP-R | 158.33 ± 2.89c | 178.33 ± 12.93bcd | 179.52 ± 15.35bcd |  | 3.40 ± 0.14b | 0.77 ± 0.02bcd |
| PS-R | 123.00 ± 2.65de | 137.09 ± 9.96de | 137.47 ± 18.00cd |  | 2.96 ± 0.11b | 0.68 ± 0.02de |
| PVC-R | 148.67 ± 34.95cd | 161.61 ± 35.51cde | 161.99 ± 33.82bcd |  | 2.23 ± 0.62c | 0.56 ± 0.11e |
| S | 204.33 ± 0.58a | 259.31 ± 11.09a | 278.15 ± 15.90a |  | 4.47 ± 0.07a | 0.82 ± 0.01abc |
| PA-S | 172.33 ± 7.23bc | 204.47 ± 23.73b | 205.00 ± 21.94b |  | 4.61 ± 0.05a | 0.91 ± 0.00a |
| PP-S | 119.33 ± 10.02e | 173.52 ± 36.65f | 173.43 ± 55.15bcd |  | 3.18 ± 0.00b | 0.73 ± 0.00cd |
| PS-S | 70.33 ± 2.89f | 89.09 ± 9.99bcde | 82.92 ± 5.59e |  | 2.98 ± 0.06b | 0.77 ± 0.01bcd |
| PVC-S | 118.00 ± 15.10e | 132.64 ± 23.73e | 130.00 ± 20.35de |  | 1.22 ± 0.57c | 0.26 ± 0.15f |
| ***Fungi*** | | | | | | |
| R | 234.33 ± 4.16a | 237.81 ± 7.49a | 239.70 ± 7.69a |  | 5.94 ± 0.02a | 0.97 ± 0.00a |
| PA-R | 113.00 ± 9.54b | 135.36 ± 15.52b | 132.56 ± 20.63b |  | 2.98 ± 0.24de | 0.80 ± 0.03b |
| PP-R | 81.00 ± 0.00cd | 91.83 ± 3.32c | 93.28 ± 6.32c |  | 3.30 ± 0.16cd | 0.85 ± 0.02b |
| PS-R | 61.67 ± 4.51e | 77.42 ± 6.92c | 79.11 ± 6.83cd |  | 2.80 ± 0.03e | 0.79 ± 0.00b |
| PVC-R | 36.67 ± 7.09f | 47.50 ± 15.82d | 47.22 ± 16.67e |  | 1.50 ± 0.14g | 0.57 ± 0.04c |
| S | 91.00 ± 9.54c | 96.05 ± 10.26c | 95.67 ± 11.73c |  | 3.68 ± 0.19bc | 0.83 ± 0.02b |
| PA-S | 82.33 ± 10.26cd | 85.77 ± 11.33c | 85.04 ± 10.13c |  | 3.73 ± 0.46b | 0.86 ± 0.08b |
| PP-S | 73.33 ± 9.45de | 82.48 ± 11.45c | 95.58 ± 17.85c |  | 2.00 ± 0.10f | 0.58 ± 0.03c |
| PS-S | 38.00 ± 9.54f | 51.34 ± 16.68d | 54.34 ± 21.37de |  | 1.45 ± 0.11g | 0.50 ± 0.04c |
| PVC-S | 41.33 ± 10.02f | 52.25 ± 13.23d | 51.23 ± 14.41de |  | 0.81 ± 0.13h | 0.28 ± 0.06d |

*R and S represented the riverine and sludge microbe inoculums, respectively. The R and S inoculated MPs-DOMs were abbreviated as XX-R and XX-S, where XX referred to the MPs-DOMs types. For example, PA-R can be interpreted as riverine microbes inoculated PA-DOM. Different lowercase letters represent significant differences between different treatments (*p* < 0.05).
